# Impact of Bioreactor Environment and Recovery Method on the Profile of Bacterial Populations from Water Distribution Systems

**DOI:** 10.1371/journal.pone.0133427

**Published:** 2015-07-21

**Authors:** Xia Luo, Kristen L. Jellison, Kevin Huynh, Giovanni Widmer

**Affiliations:** 1 Lehigh University, Department of Civil and Environmental Engineering, 1 W. Packer Avenue, Bethlehem, Pennsylvania, United States of America; 2 Cummings School of Veterinary Medicine at Tufts University, Department of Infectious Disease and Global Health, North Grafton, Massachusetts, United States of America; Catalan Institute for Water Research (ICRA), SPAIN

## Abstract

Multiple rotating annular reactors were seeded with biofilms flushed from water distribution systems to assess (1) whether biofilms grown in bioreactors are representative of biofilms flushed from the water distribution system in terms of bacterial composition and diversity, and (2) whether the biofilm sampling method affects the population profile of the attached bacterial community. Biofilms were grown in bioreactors until thickness stabilized (9 to 11 weeks) and harvested from reactor coupons by sonication, stomaching, bead-beating, and manual scraping. High-throughput sequencing of 16S rRNA amplicons was used to profile bacterial populations from flushed biofilms seeded into bioreactors as well as biofilms recovered from bioreactor coupons by different methods. β diversity between flushed and reactor biofilms was compared to β diversity between (i) biofilms harvested from different reactors and (ii) biofilms harvested by different methods from the same reactor. These analyses showed that average diversity between flushed and bioreactor biofilms was double the diversity between biofilms from different reactors operated in parallel. The diversity between bioreactors was larger than the diversity associated with different biofilm recovery methods. Compared to other experimental variables, the method used to recover biofilms had a negligible impact on the outcome of water biofilm analyses based on 16S amplicon sequencing. Results from this study show that biofilms grown in reactors over 9 to 11 weeks are not representative models of the microbial populations flushed from a distribution system. Furthermore, the bacterial population profile of biofilms grown in replicate reactors from the same flushed water are likely to diverge. However, four common sampling protocols, which differ with respect to disruption of bacterial cells, provide similar information with respect to the 16S rRNA population profile of the biofilm community.

## Introduction

Biofilms in water distribution systems can negatively impact water quality. Most of the biomass in distribution systems is attached to pipe surfaces in a biofilm, with > 98% of total bacteria associated with the pipe wall biofilm and loose deposits [1]. Analysis of water distribution pipes by scanning electron microscopy has confirmed the presence of biofilms on pipe surfaces and in tubercles. Adherent microbial communities may increase water turbidity or cause taste, color and odor problems [2], harbor pathogenic microorganisms [3–7], and cause accelerated corrosion of some types of pipes [8].

Sampling biofilms in water distribution systems is difficult because of limited access to buried, pressurized water mains. Most studies that report data from direct sampling of biofilms on the surfaces of water mains have collected samples while a leaking or burst pipe was being repaired or during otherwise scheduled maintenance work. Water main biofilms must be sampled when the main is accessible, which is not likely to frequently overlap with times when watershed conditions, finished water quality, or consumer health concerns indicate that sampling should be done. In addition, accessible water mains due to a burst pipe or routine repairs/maintenance are susceptible to abrupt changes in water flow and shear stresses, as well as environmental contamination. Thus, sampling biofilms in water main at these times may not provide data that are representative of microbial activity under normal flow conditions.

Challenges associated with direct sampling of biofilms in distribution systems could be circumvented if biofilms flushed from the water distribution system and analyzed in the laboratory were proven to be representative of the adherent microbial composition in the largely inaccessible water mains. In this study, we used rotating annular reactors to grow biofilms from biomaterial flushed from two water distribution systems (in California and Pennsylvania, respectively) to assess whether the microbial community composition in the bioreactors is representative of the flushed distribution system biomaterial. Rotating annular reactors are particularly popular for studying biofilms in the laboratory because they enable the modeling of shear stress conditions experienced by biofilms in water distribution systems [9].

Whether biofilms are sampled directly from distribution systems or grown in bioreactors, the impact of the sampling method on recovery of biomaterial has not been assessed until now. Reliable characterization of attached biomass may be hindered by strongly adherent microbial cells. Efforts to detach those cells may have unintended impacts on downstream analyses. Therefore, understanding the impact of the initial biofilm sampling procedure on the resultant biomass characterization is critical to correctly interpreting the data generated by researchers and utilities studying biofilms in water distribution systems.

In the experiments presented here, we used 16S ribosomal RNA (rRNA) amplicon sequencing to compare the β diversity between bacterial populations from biofilms flushed from water distribution systems and biofilms grown in rotating annular reactors seeded with the flushed water samples. These data were analyzed to answer the following research questions: (1) Are biofilms grown in bioreactors representative of biofilms flushed from the water distribution system in terms of bacterial composition and diversity? (2) Does the biofilm sampling method affect the population profile of the bacterial community? Results from this work will assist water utilities in identifying appropriate biofilm monitoring protocols to characterize microbial communities on distribution system surfaces. The ultimate goal is routine biofilm monitoring in water distribution systems to enable timely and effective interventions to maintain treated water quality and protect consumer health.

## Materials and Methods

Biofilm samples were flushed from hydrants. Samples were collected by Golden State Water, Rancho Cordova, California, and the Department of Water & Sewer Resources, City of Bethlehem, Pennsylvania. Samples collection was performed and authorized by the respective utility. No other field work was performed as part of this study and no endangered or protected species were involved. No animal research was performed as part of this study.

### Flushed biofilms and bioreactors

Four closed-loop rotating annular bioreactors were run in parallel for each experiment (Figure A in S1 File). The working volume of each bioreactor was 450 mL. Each bioreactor consisted of a rotating inner cylinder (radius = 31.75 mm) to promote mixing and a stationary outer cylinder (i.e., closed-mouth glass jar; radius = 42.5 mm). Twelve removable polycarbonate coupons (2 mm thickness) were installed in slots on the inner cylinder. The pump was set to control flow in each bioreactor loop at approximately 210 mL/min. The rotational speed of the inner cylinder in each bioreactor was approximately 132 rpm, resulting in turbulent flow (Reynolds number = 4700, calculated according to Dou et al. [10]) and a shear stress of 0.063 Pa at the inner wall [11]. The reactors were operated in aerobic conditions.

Two replicate experiments were performed using biofilms flushed from the Golden State Water (Rancho Cordova, California) and the city of Bethlehem, Pennsylvania water distribution systems. Bioreactors were seeded with biofilm material from each utility to assess the applicability of our results to waters from different geographic locations that have been exposed to different types of disinfectants (Golden State Water has a chloramine residual, while the city of Bethlehem has a chlorine residual).

### Water quality measurements

Bioreactors were run for 11 weeks for the experiment with flushed Golden State water and nine weeks for the experiment with Bethlehem water. For the duration of the experiments, bioreactors were monitored weekly to assess changes in biofilm thickness and water quality (S1 File). To measure water quality, 158 mL (35% of the total water volume) was removed weekly from each reactor in the first experiment (Golden State water) and 283 mL (63%) was removed weekly in the second experiment (Bethlehem water) and replaced with flushed water stored at 4°C. Water quality tests were also performed on the flushed stored Golden State water (at the beginning of the experiment only) and Bethlehem water (at the beginning of the experiment and weekly throughout the duration of the experiment).

### Scanning confocal laser microscopy

After biofilm thickness stabilized, two coupons from each bioreactor were imaged by scanning confocal laser microscopy (SCLM). Biofilms were fixed and stained with Syto 9 (Life Technologies) as described previously [4]. Fluorescent z-stack images were captured with a Zeiss LSM 510 inverted scanning-laser confocal fluorescence microscope (25x NA = 0.8, H_2_O immersion, Carl Zeiss, Jena, Germany) equipped with a 488-nm argon laser. The image analysis program COMSTAT [12] was used to quantitatively analyze these z-stack images for average thickness, bio-volume, total surface area, surface area-to-volume ratio, and roughness coefficient. Details of COMSTAT analysis of SCLM images are presented in S1 File.

### Coupon processing and recovery of biofilms

Of the 12 coupons in each bioreactor, 8 were sampled by four methods (2 coupons for each method; details provided below) and analyzed by flow cytometry and 16S amplicon sequencing; two were imaged with SCLM (details provided above) and two were used for analysis of mass of volatile suspended solids (Golden State experiment, data not shown) or mean biofilm thickness (Bethlehem experiment).

Biofilms grown on bioreactor coupons were recovered using four methods: manual scraping, stomaching, sonication and bead beating. For recovering biofilm by manual scraping, two coupons from each bioreactor were removed with sterile tweezers; each coupon was scraped with a sterile cell scraper (3 cm blade, BD Falcon, BD Biosciences, San Jose, California) and rinsed several times with ultrapure Millipore water until the surface appeared clear. Cell scrapings and rinse water from the same coupon were collected in a sterile 50-mL conical centrifuge tube and vortexed for 30 s.

For recovering biofilm by stomaching, each coupon was transferred into a sterile stomacher bag (102 × 152 mm) containing approximately 30 mL of Millipore water. After about 30 min on the benchtop, the stomacher bag was inserted into the stomacher (Stomacher 80 Biomaster, Seward, London, UK) and the sample was blended at 230 rpm for 2 min. The contents of the stomacher bag were then transferred to a sterile 50-mL conical centrifuge tube.

For recovering biofilm by sonication, two coupons were each transferred to a separate sterile container and immersed in ultrapure water. After about 15–25 min on the benchtop, the sonicator probe was inserted into the water about 1 cm above the coupon surface. Gentle sonication was performed with 2 min of ultrasound at 2 W and 20 KHz. During sonication, the sonicator probe was moved along the length of the coupon to ensure that the entire coupon surface was processed. The coupon was removed from the container aseptically, and the water in the container was transferred to a sterile 50-mL centrifuge tube.

For recovering biofilm by bead beating, two coupons from each bioreactor were cut into 18–20 small pieces (about 0.6 x 1.2 cm) with sterile scissors, avoiding as much as possible disturbance to the biofilm. Two to three small pieces of the same coupon were transferred to a 1.5 mL screw-capped microcentrifuge tube with 0.6 g of autoclaved 0.5 mm zirconia/silica beads. One mL of ultrapure water was added to the microcentrifuge tube, and the tube was placed in the bead beater (Mini-BeadBeater-8, BioSpec Inc.) for two 1-min cycles with a 2-min incubation on ice in between cycles. After the second cycle, the tube was cooled on ice for 2 min, and the liquid in the tube was transferred to a sterile 50-mL centrifuge tube.

### Flow cytometry

We used flow cytometry to assess the extent of disruption biofilm cell disruption. Particle (cells and debris) concentration in reactor biofilms samples recovered from the coupons by four different methods (see previous section) were analyzed. To avoid clogging the cytometer, biofilm samples were sieved through a 40-μm pore size Nylon strainer. A total of 10,000 events were counted using a forward scatter threshold of 80,000, corresponding to approximately 1.9 μm using a C6 Accuri flow cytometer (BD Biosciences). The scatter signal was calibrated using a set of six calibration beads ranging in diameter from <2 μm to 15 μm.

### 16S amplicon sequencing, bioinformatics and data analysis

Biofilm samples flushed from the distribution system and recovered from coupons as described above were suspended in 200 μL water and subjected to two cycles of freeze-thawing (-80°C/37°C) to lyse the cells and facilitate DNA extraction. The protocol for generating amplicon libraries for high-throughput sequencing was previously described [13]. DNA extracted with the HighPure PCR Template Preparation kit (Roche Diagnostics) was suspended in approximately 200 μL of water. 16S amplicon libraries were prepared from the V6 region (first experiment with Golden State water) or the V1V2 region (second experiment with Bethlehem water) of the 16S rRNA gene. All samples from a same experiment were analyzed using the same 16S rRNA region. Additional details are found in S1 File.

## Results and Discussion

### Biofilm development and water quality parameters

In both experiments, water quality in each of the four bioreactors was similar (A and B Tables in S1 File). The similarity is likely explained by the fact that circulating water was refreshed weekly using the same stock of flushed water as used to seed the reactors. This stock was stored refrigerated in the dark. Total nitrogen concentration decreased gradually in the bioreactors run with Golden State water. Because the stored composite water was tested only at the beginning of the Golden State experiment, the observed decrease in total nitrogen may have resulted from decreasing nitrogen concentration in the stored water. The concentration of NH_3_-N in stored composite Bethlehem water was measured weekly and found to be greater than the concentration measured in the bioreactors (B Table in S1 File), suggesting that nitrogen was consumed in the bioreactors to support biofilm growth. For the bioreactors run with flushed Bethlehem water, the concentration of TOC in the bioreactors was greater than that in the composite water and also decreased over time. The excess TOC in the bioreactor water in the early weeks of the experiment was attributed to residual ethanol remaining in the bioreactor system after sanitizing the tubing between experiments.

**Fig 1:**
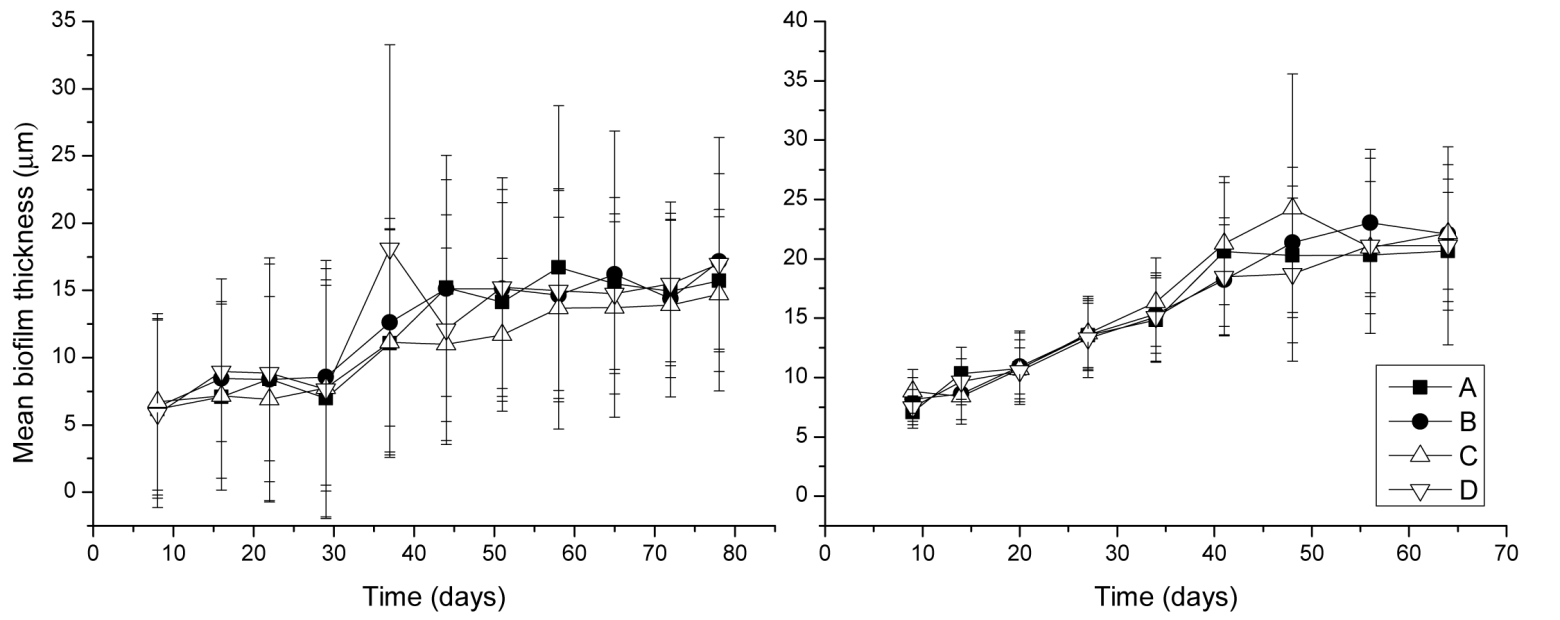
Mean biofilm thickness measured using light microscopy as a function of time in bioreactors A, B, C, and D for experiment with flushed Golden State water (left) and flushed Bethlehem water (right), respectively. Error bars indicate one standard deviation.

Weekly biofilm thickness measurements (Fig 1) showed that thickness stabilized after approximately seven weeks. C Table in S1 File and Figure B in S1 File report biofilm characteristics computed by COMSTAT analysis of SCLM z-stack images as described in S1 File. Biofilms grown with the Bethlehem water generally had a 13% greater surface area than biofilms grown with the Golden State water, although this difference was not statistically significant. The surface area-to-volume ratio of biofilms grown from flushed Golden State water was significantly lower (unpaired t-test, two-tail, *p* = 0.0045) than that of biofilms grown from flushed Bethlehem water. This difference might be explained by the relatively lower phosphorus and nitrogen levels in the Bethlehem water (A and B Tables in S1 File). Biovolume and biofilm thickness followed similar trends, with Golden State biofilms generally 19.6% thicker and with 37.9% higher calculated biovolume than Bethlehem biofilms. Significant variations (one-way ANOVA, *p* < 0.01 for both Golden State water and Bethlehem water) in roughness of bioreactor biofilms grown from the same flushed water were recorded. There was no significant difference between the mean biofilm thickness measured by light microscopy (Fig 1) and by SCLM (C Table in S1 File).

### Flow cytometry

Particle concentration in biofilm samples recovered with each of the four methods described above was measured to assess the impact of each method on the integrity of biofilm microorganisms. As expected, bead beating resulted in the largest particle concentration. An average of 4271 events/μL (standard deviation (SD) 2537) was measured in 8 replicate biofilm samples recovered from coupons using this method. In order of decreasing particle concentration, stomaching generated a mean of 1954 events/μL (n = 8, SD 1075), scraping produced in average 1574 events/μL (n = 8, SD 887) and sonication 580 events/μL (n = 8, SD 813). ANOVA on ranks indicated that the effect of recovery method on particle concentration was significant (H = 18.3, 3 d.f., p<0.001). These results were obtained with biofilm grown from flushed Golden State water.

### Analysis of biofilm bacterial communities

**Fig 2:**
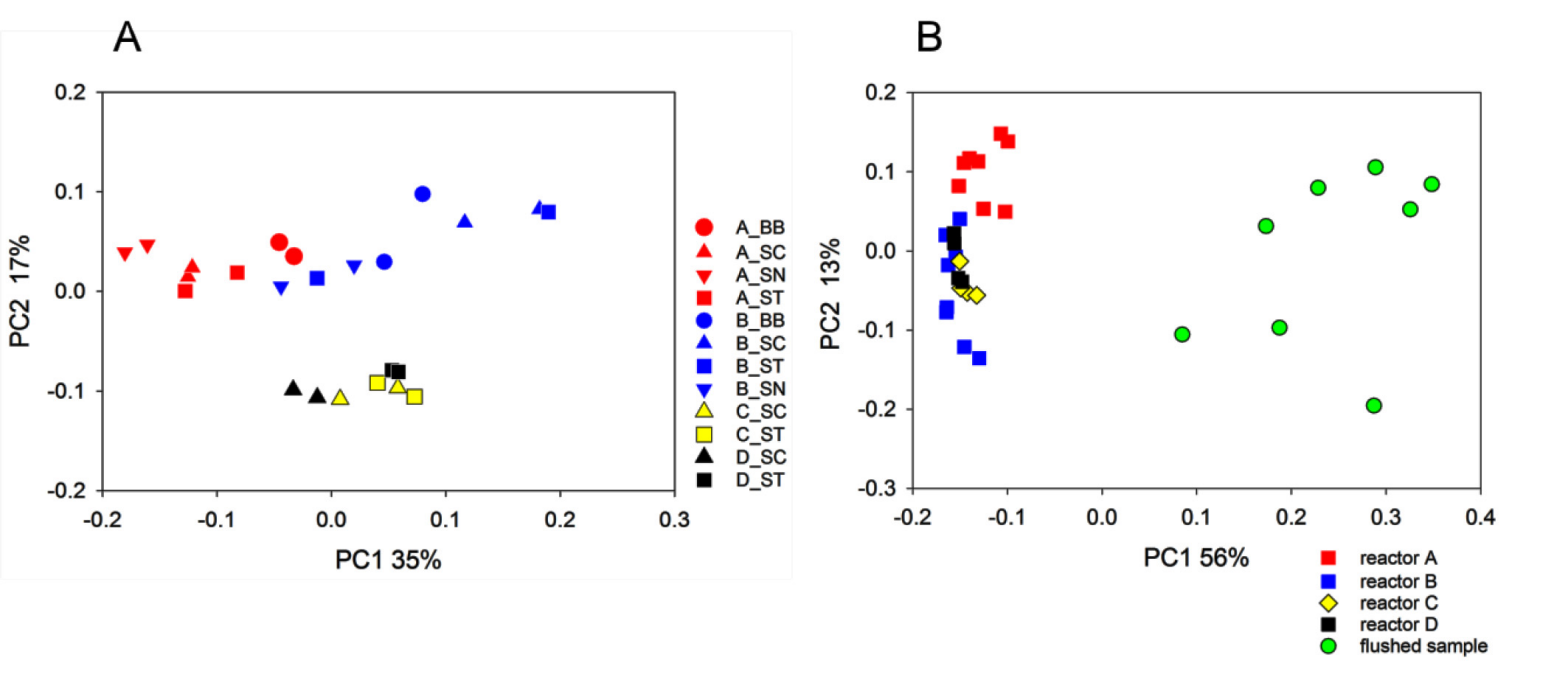
Principal Coordinate Analysis of reactor biofilms grown from flushed Golden State biofilms. A, 24 biofilm samples are represented according to biofilm recovery method (symbol) and reactor (color). The key indicates reactor_method, where circle = bead beating (BB), triangle up = scraping (SC), triangle down = sonication (SN), and square = stomaching (ST). B, PCoA illustrating distances between flushed (n = 8) and reactor biofilms. Distances between symbols are indicative of phylogenetic (Unifrac) distance. Percent variation explained by each axis is indicated.

We compared the bacterial community in flushed Golden State biofilm to steady-state biofilms recovered from annular reactors seeded with this biofilm using 16S amplicon sequencing. A total of 275,530 sequences screened as described in the S1 File were used. Principal Coordinate Analysis (PCoA) based on pairwise weighted Unifrac distances between biofilms was used to visualize the dissimilarity among bacterial populations according to recovery method and reactor (Fig 2). Distances between data points in PCoA plots are an approximate representation of phylogenetic dissimilarity. This analysis shows that biofilms clustered according to reactor; in particular, A and B biofilms formed two distinct clusters whereas C and D biofilms formed a single cluster. The statistical significance of clustering was assessed using ANOSIM [14]. Consistent with the PCoA plots, clustering by reactors was highly significant (R = 0.65, p<0.0001). According to ANOSIM, clustering was not impacted by the method used to recover biofilms (R = 0.12, p = 0.07). The 16S sequence data thus indicate that bacterial communities in biofilms grown in different bioreactors diverged from each other and that the recovery method had no significant impact on the 16S data.

Bacterial microbiota from biofilms grown in reactors for 11 weeks diverged from their original profile in the flushed biofilm from the Golden State Water system (Fig 2, S1 Table and Figure C in S1 File). The bioreactors were seeded with a mixture of 8 samples flushed from different hydrants in the Golden State distribution system (represented with green circles in Fig 2). We used rarefaction analysis [15] to compare the overall richness of bacterial populations from the reactor biofilms versus bacterial populations flushed from hydrants. This analysis (not shown) revealed that most biofilms grown in reactors were more diverse than those from the flushed samples. The Shannon and Simpson α diversity indices were consistent with rarefaction analysis. Of a total of 32 samples (8 flushed, 24 reactors), the 6 least diverse samples were flushed and the 13 (16 according to Simpson index) most diverse samples were from bioreactors (Mann-Whitney Rank Sum test, p<0.001 for both diversity indices). As indicated by PCoA (Fig 2) and the variance partition analysis described below, Shannon and Simpson diversity analyses revealed no difference among recovery methods (Kruskal-Wallis ANOVA on ranks, p = 0.09 for Shannon, p = 0.66 for Simpson).

**Fig 3:**
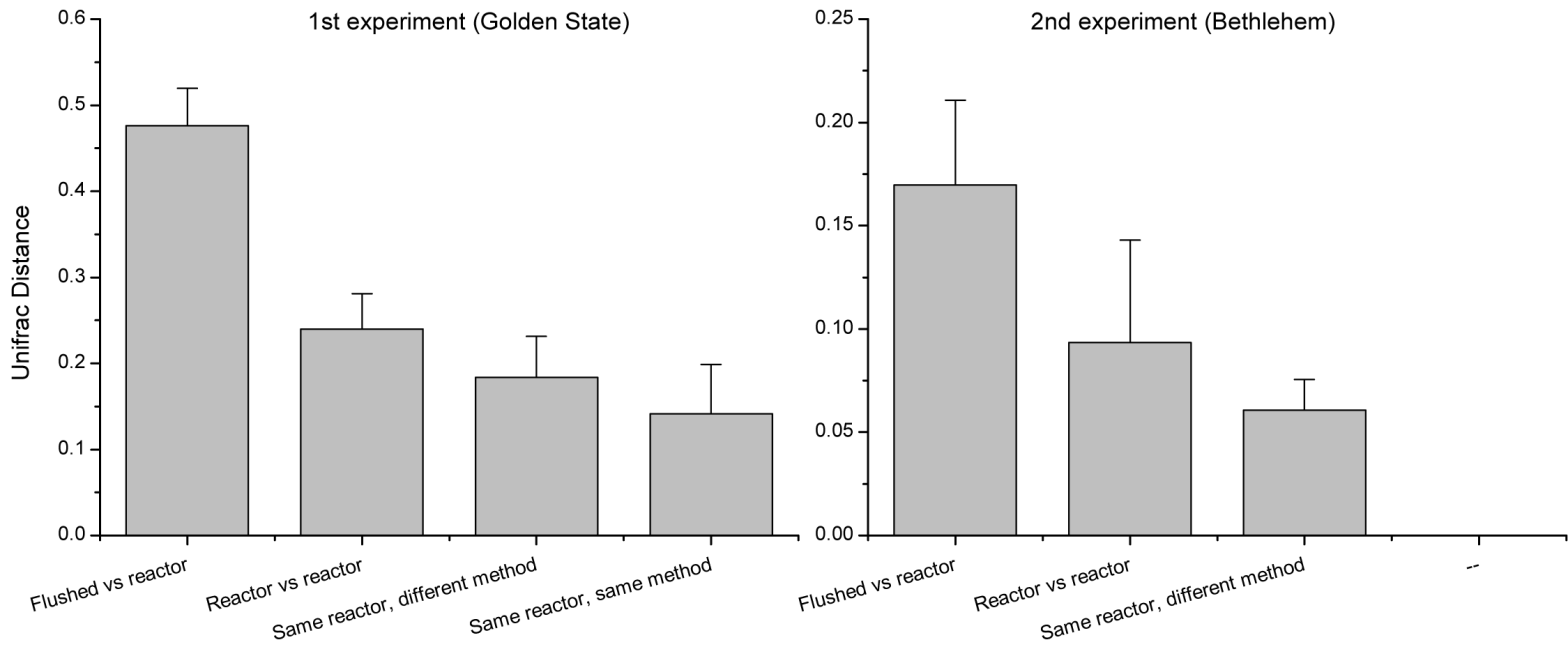
Mean β diversity between bacterial populations from flushed water and recovered from reactor biofilms. Mean Unifrac distance between flushed and bioreactor biofilms exceeds distance between biofilm grown in different reactors and biofilms recovered using different methods.

To assess the effect of the main experimental variables on biofilm bacterial populations, we compared mean Unifrac distances (D values) between flushed and bioreactor bacterial populations for four bioreactors (192 distance values), between bacterial populations grown in different bioreactors (208 distance values), between bacterial populations from the same bioreactor recovered with different methods (56 distance values), and between bacterial populations from the same bioreactor recovered by the same method (12 distance values) (Fig 3). Consistent with PCoA and ANOSIM, the average distance between flushed and bioreactor samples was the largest (D = 0.48, SD = 0.04), followed by the mean distance between biofilms grown in different bioreactors (recovered by any method) (D = 0.24, SD = 0.04), the mean distance between biofilms from the same bioreactor recovered by different methods (D = 0.18, SD = 0.04), and the mean distance between biofilms from the same bioreactor recovered by the same method (D = 0.14, SD = 0.5). Since biofilms recovered by the same method from the same reactor are experimental replicates, the mean distance between these biofilms represents a measure of experimental noise. Thus, we tested which comparisons were significantly larger than the experimental noise. An ANOVA on ranks showed that all comparisons exceeded experimental noise, except the distances between bioreactor biofilms recovered from the same bioreactor using different methods. These results were confirmed by a Variation Partitioning Analysis [16] comparing the relative impact of bioreactor and biofilm recovery method on bacterial population profile. According to this analysis, the reactor effect explained 2.9 times more variation than the recovery method.

The observed difference between the flushed microbial community and the biofilms grown in the bioreactors indicates that reactor biofilms may not have reached full maturity, even though biofilm thickness had stabilized [17] (Fig 1). These results, together with the flow cytometric characterization of recovered biofilm material described above, also indicate that the extent of cell disruption does not majorly impact 16S sequence data. It is understood that other variables such as sample processing, DNA extraction procedure, and 16S PCR primer specificity can impact the results of biofilm analyses, but in our context these variables are not relevant, as the same methods were applied to all samples from the same experiment.

The taxonomy of the flushed and bioreactor biofilms was investigated to assess which bacterial taxa changed significantly when comparing flushed and bioreactor samples. Taxa differing significantly in abundance were identified using program LEfse [18] (S1 Table). A sequence classified in the genus *Rheinheimera* (class *Gammaproteobacteria*) represented 29% (13145/45282) of the sequences identified as significantly over-represented in flushed biofilms as compared to biofilms grown in reactors. The most abundant over-represented sequence in the reactor biofilms was unclassified at a probability level of 70%. This sequence represents 28% (39459/140122) of sequences over-represented in reactor biofilms.

**Fig 4:**
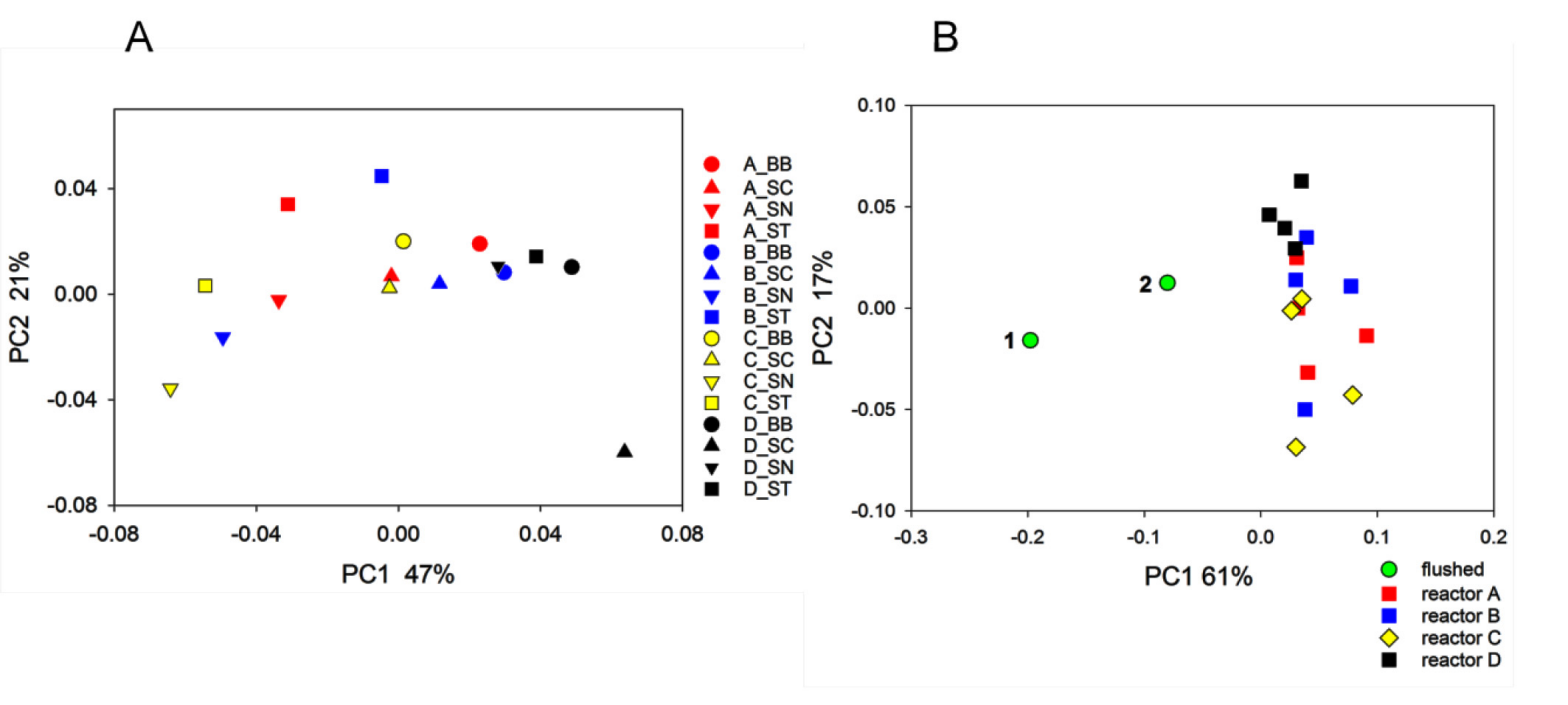
Principal Coordinate Analysis of reactor biofilms grown from flushed Bethlehem distribution system biofilms. A, 16 biofilm samples are represented according to biofilm recovery method (symbol) and reactor (color). The key indicates reactor_method, where circle = bead beating (BB), triangle up = scraping (SC), triangle down = sonication (SN), and square = stomaching (ST). B, PCoA illustrating distances between flushed (n = 2) and corresponding reactor biofilms. Flushed biofilm 1 indicated in plot B was seeded into the reactors. Sample 2 is a second sample flushed from the same hydrant a few seconds later. Distances between symbols are indicative of phylogenetic (Unifrac) distance. Percent variation explained by each axis is indicated.

The bioreactor experiment described above was replicated with flushed biofilm samples collected from the Bethlehem water distribution system. A total of 166,062 sequences screened as described in S1 File were used. Fig 4 shows a PCoA of 16 biofilms recovered from four bioreactors by bead beating, scraping, sonicating, and stomaching. With the exception of reactor D samples, which formed a distinct cluster, little divergence between bioreactor A, B and C samples was observed, indicating that reactor did not impact biofilm composition to the extent observed in the first experiment. This interpretation is supported by statistical analysis with ANOSIM which detected borderline significant clustering when all reactors were included (n = 4, R = 0.19, p = 0.044). As expected, reactors A and D were significantly different from each other (R = 0.43, p = 0.026), as were reactors C and D (R = 0.39, p = 0.028). The other pairwise comparisons were not significant. A combined analysis of 16 bioreactor samples and two flushed biofilms used to seed the reactors is also shown in Fig 4. The two samples of flushed water were collected from the same hydrant within a few seconds from each other. As observed in the first experiment, the bioreactor biofilms diverged significantly from the flushed water samples. The mean Unifrac distance D between flushed biofilm samples and bioreactor samples (32 D values) was 0.17 (SD = 0.04), whereas biofilms grown in different bioreactors were separated on average by a D value of 0.09 (n = 121, SD = 0.05). The mean distance between biofilms from the same bioreactor recovered by different methods was 0.06 (24 comparisons, SD = 0.02) (Fig 3). For this experiment, we were unable to estimate the magnitude of the experimental error because of the lack of replicates, i.e., samples recovered from the same reactor using the same method. As in the first experiment, all comparisons were statistically significant (ANOVA on ranks, p<0.05). Although absolute distances were larger in the Golden State experiment, relative distances for the different comparisons were similar across the two experiments. As a percent of flushed vs. reactor distance, the distance between biofilms from different reactors equaled 50% and 54%, respectively, in the Golden State and Bethlehem experiments, whereas the distance between biofilms from the same reactor recovered by different methods equaled 30% and 35%, respectively.

With respect to taxonomy in the experiment with flushed Bethlehem water, the relative abundance of *Alphaproteobacteria* (Order *Rhizobiales* in particular) significantly increased in the bioreactors. Whereas in the flushed sample *Alphaproteobacteria* represented <10% of the population, in the bioreactors they represented 36% of the sequences (p = 0.02 by LEfse analysis; D Table in S1 File and S2 Table, Figure C in S1 File).

### Integration of water quality and sequence data

When contrasting PCoA plots derived from water quality data (S1 File) with PCoAs based on16S sequences pooled by reactor (not shown), a similarity between water quality and sequence data was noticed. We used Procrustes analysis [19] to quantify for each experiment the similarity between water quality and 16S data. The Disagreement values [20] between water quality and 16S data for Golden State and Bethlehem experiments were 0.17 and 0.11, respectively, where a value of 0 indicates perfect agreement between reactor scores (i.e., the location of data points on a plot) and a value of 1 indicates maximal disagreement. To put these numbers in perspective, water quality and 16S data were paired across experiments, i.e., Golden State water quality with Bethlehem 16S data and vice versa. This artificial analysis yielded Disagreement values of 0.19 and 0.34. Although additional randomizations would be needed to assess statistical significance, the lower disagreement obtained with matched data sets suggests a correlation between water quality and biofilm bacterial community. Given that the water circulating in each reactor was refreshed weekly using water from a common stock, we tentatively assume that these observations indicate that divergence of biofilms grown in different reactors impacts water quality and not vice versa. These results emphasize the need for replication, as biofilms growing in parallel reactors may diverge.

## Conclusions

- This study has important implications for water utilities seeking to adopt protocols for assessing microbial growth in water distribution mains and laterals.
- Biofilms grown in reactors over 9 to 11 weeks are not representative models of the microbial populations flushed from a distribution system, and therefore are not predictive of conditions within the distribution system.
- 16S amplicon sequencing of bacterial microbiota grown in rotating annular reactors operated in parallel revealed a significant divergence of water quality and biofilms in different reactors.
- Four common sampling protocols which differ with respect to disruption of bacterial cells provide similar information on biofilm composition.
- The similar outcome of the two bioreactor experiments seeded with different flushed biofilms and analyzed using different 16S variable regions indicates that our results are generally applicable.

## Supporting Information

S1 File Supporting Methods and Results. (DOCX)

S1 Table Taxonomy of V6 sequences differentially represented between biofilm flushed from eight sites in the Golden State distribution system and biofilms recovered from four bioreactors. (PDF)

S2 Table Taxonomy of V1V2 sequences differentially represented between biofilm flushed from the Bethlehem distribution system and biofilms recovered from four bioreactors. (PDF)
