# Supplementary material for: Impact of Bioreactor Environment and Recovery Method on the Profile of Bacterial Populations from Water Distribution Systems
Source: PLoS One. 2015 Jul 21;10(7):e0133427. doi: 10.1371/journal.pone.0133427 (PMC4509647; doi:10.1371/journal.pone.0133427)
Supplement: S1 File — (DOCX) [file pone.0133427.s001.docx]

**Supporting Information**

**Methods**

**Bioreactors and experimental design**

Flushed water from the Golden State distribution system was collected and shipped overnight to Lehigh University; flushed water from the Bethlehem distribution system was immediately transported to the laboratory. Once in the laboratory, flushed water was stored in a 20-L carboy in a 4°C cold room in the dark. Bioreactors were filled with this flushed water at the start of each experiment. Once per week, flushed water from the stored carboy was agitated for approximately 2 min and used to refresh the water flowing in the bioreactors. Bioreactors were operated under recirculating conditions because it was not logistically feasible to collect enough water to run the reactors in flow-through mode for the duration of the experiments. Therefore, bioreactors were operated in recirculating mode with careful monitoring to ensure that water quality conditions did not change significantly in the time between weekly bioreactor water changes. This approach is similar to one used by Lawrence et al. [[1](#_ENREF_1)] to grow river biofilms in annular reactors run in recirculating mode with water changed on an 8-day cycle.

Six coupons (one from each bioreactor and a duplicate coupon from two bioreactors) were temporarily removed weekly for nondestructive biofilm thickness measurements by light microscopy, using the stage micrometer of a Nikon ECLIPSE 50i optical microscope according to a protocol adapted from Trulear and Characklis [[2](#_ENREF_2)]. Briefly, biofilm thickness along the center transect of the coupon in a total of 100 focal circles was measured. The 40× objective was lowered until the top of the biofilm surface was in focus, and the fine adjustment dial setting of the stage micrometer was recorded. The objective was then lowered until the inert polycarbonate coupon surface was in focus and the dial setting again recorded. The difference in fine adjustment settings equaled the biofilm thickness (each gradation on the fine focus adjustment dial equals a distance of one micron). Average biofilm thickness was recorded as the mean value of the 100 thickness readings per coupon. Bioreactor growth was terminated and coupons were removed for recovery of biofilms once the average biofilm thickness was observed to stabilize.

**SCLM images analyzed by COMSTAT**

COMSTAT analyzes individual voxels with the dimensions (pixel size)x × (pixel size)y × (pixel size)z. The biovolume is defined as the number of biomass pixels in all images of a z-stack multiplied by the voxel size and divided by the substratum area (i.e., the area colonized by biofilm in the bottom image of the z-stack). The biovolume reflects how efficiently the substratum is colonized by bacteria; a given volume of biomass that grows from a smaller footprint area would have a higher calculated biovolume (and be thicker) than the same volume of biomass spread out over a larger footprint. The surface area is the area summation of all biomass voxel surfaces exposed to the background. The surface area-to-volume ratio reflects the fraction of the biofilm that is exposed to the nutrient flow. Microbial communities adapt to their environments, and therefore, in low nutrient environments, the surface area-to-volume ratio would be expected to increase to optimize access of the biofilm community to the limited supply of nutrients. The roughness coefficient quantifies variation in the biofilm thickness and also provides an indication of biofilm heterogeneity; a lower roughness coefficient indicates a more uniform and smooth biofilm culture.

**Water quality measurements**

Water from the stored composite water and bioreactors was analyzed for temperature; pH and conductivity (Oakton Ion 510 benchtop meter, Vernon Hills, IL, USA); turbidity (HACH 2100AN turbidimeter, Loveland, CO, USA); hardness (HACH Method 8204); alkalinity (HACH Method 8203); total organic carbon (Bethlehem water only, Shimadzu TOC analyzer V_CPH_ model, Kyoto, Japan); total nitrogen (Golden State water only, Shimadzu TOC analyzer with the TNM-1 nitrogen module); nitrate-N, nitrite-N, and ammonia-N (Bethlehem water only, HACH Method 8171, 8507, and 8155, respectively); and phosphorus (HACH Method 8048).

**16S amplicon libraries and bioinformatics**

DNA extracted as described in METHODS was dissolved in 20 µl of Tris-HCl pH 8.5. A volume of 1 µL of DNA was amplified in a primary PCR with primers flanking the approximately 60-nucleotide variable V6 region of the bacterial 16S rRNA gene [[3](#_ENREF_3)]. The following primers were used in the primary PCR: 5’-CAACGCGAAGAACCTTACC-3’ and 5’-CGACAGCCATCGANCACCT-3’. The primary amplification consisted of 15-25 cycles of 94°C for 30 s, 55°C for 30 s, and 68°C for 90 s. A second PCR was used to incorporate Illumina adapter sequences and unique 6-nucleotide barcodes. Amplicon libraries were sequenced in an Illumina HiSeq2000 sequencer (Illumina, San Diego, California) at the Tufts Medical School Genomics Core (tucf.org).

To take advantage of the capability of the MiSeq instrument to generate longer reads, the second experiment was analyzed using sequences from the V1V2 region of the 16S rRNA gene. We adopted the same conserved 27F and 338R primers used by others [[4](#_ENREF_4), [5](#_ENREF_5)], where the sequence of 27F is 5’-AGAGTTTGATYMTGGCTCAG-3’ and the sequence of 338R V2 is 5’-TGCTGCCTCCCGTAGGAGT-3’. The length of the V1V2 amplicon is approximately 350 nucleotides. Illumina adaptor sequences and a unique 6-nt barcode were incorporated into the amplicon during a secondary PCR. Random subsamples of 10,000 V6 and V1V2 sequences, respectively, were analyzed per sample.

For each biofilm sample, random subsamples of 10,000 sequences were analyzed. A bioinformatics pipeline based mainly on programs found in *mothur* [[6](#_ENREF_6)] was used to curate, classify and cluster sequences. Briefly, sequence reads were aligned using Clustal Omega [[7](#_ENREF_7)] and curated to remove sequencing errors by eliminating sequences with one or more ambiguous nucleotide calls and homopolymers longer than 8 nt. Chimeras were removed using Uchime [[8](#_ENREF_8)] as implemented in *mothur*. Sequences which did not align or were significantly shorter or longer than the majority were removed. Differences in the profile of the bacterial populations in different samples were quantified using the weighted Unifrac phylogenetic distance D [[9](#_ENREF_9)]. D ranges for 0 to 1, where 0 indicates identical populations and 1 indicates the absence of shared taxa. Matrices of pairwise distances were computed in *mothur* and visualized on Principal Coordinate Analysis (PCoA) plots using GenAlEx [[10](#_ENREF_10)]. Taxa differing significantly in abundance were identified using program LEfse [[11](#_ENREF_11)]. Sequences were classified using the Naïve Bayesian classifier [[12](#_ENREF_12)] with template and taxonomy reference files downloaded from the Ribosomal Database Project [[13](#_ENREF_13)]. Rarefaction analysis [[14](#_ENREF_14)] was performed with EstimateS software (http://viceroy.eeb.uconn.edu/estimates/) and used to compare sequence diversity for equal sequencing effort. Sequences were submitted to the European Nucleotide Archive under accession number PRJEB8234.

We applied Variation Partitioning Analysis (Borcard *et al.* 1992) as implemented in CANOCO [[15](#_ENREF_15)] to experiment 1 sequence data to assess the relative contribution of reactor and biofilm recovery method to variation in OTU abundance among 24 bioreactor samples from the first experiment. Reactor and recovery method were used as independent variables and the 200 most abundant OTUs as dependent variables. A simple effect model was used. Data from the second experiment were not analyzed because of an insufficient number of samples (n=16) in relation to the number of reactor × recovery method combinations (n=16).

Procrustes analysis [[16](#_ENREF_16)] was used to assess for each experiment the similarity between PCoA plots based on water quality and bacterial population profile. The dissimilarity between the quality of the water circulating in the reactors was calculated by normalizing mean water quality data recorded for each reactor. In the first experiment with biofilms from the Golden State distribution system, nine water quality parameters were measured (S1 Table), whereas in the second experiment with Bethlehem flushed samples, 13 water quality parameters were measured (S2 Table). Normalization by variable was performed by subtracting the mean and dividing by the standard deviation, and the dissimilarity among reactors expressed as Euclidean distance. The Bray-Curtis distance metric was used to quantify the difference between biofilm bacterial populations in each reactor. For this analysis, 16S sequences from different coupons obtained from the same reactor were pooled. Water quality and 16S PCoA plots were then compared with Procrustes and the disagreement between PCoA plots expressed as Disagreement Value calculated as described [[15](#_ENREF_15)].

**Table** **S1. Water quality for flushed Golden State water (baseline) and 11-week bioreactor averages**

| Water Quality Parameter^a^ | Baseline  (6/8/12) | Bioreactor | | | |
| --- | --- | --- | --- | --- | --- |
|  |  | A | B | C | D |
| Temperature (℃)^a^ | 24 | 26.1 ± 1.48 | 25.4 ± 1.18 | 24.9 ± 1.06 | 24.3 ± 0.83 |
| pH^b^ | 7.02 | 7.85 ± 0.42 | 7.97 ± 0.32 | 7.99 ± 0.21 | 8.03 ± 0.18 |
| Conductivity (µs/cm) ^b^ | 576 | 366 ± 98.81 | 394 ± 106.59 | 350 ± 114.77 | 357 ± 120.87 |
| Hardness ^a^  (mg/L as CaCO_3_) | 177 | 102 ± 10.05 | 104 ± 2.77 | 98 ± 4.94 | 98 ± 7.18 |
| Turbidity^b^  (NTU) | 20.967 | 11.05 ± 3.32 | 7.45 ± 1.87 | 7.57 ± 3.41 | 7.69 ± 3.82 |
| Phosphorus^b^  (mg/L as PO_4_^3-^) | 1.37 | 0.80 ± 0.509 | 0.79 ± 0.265 | 0.64 ± 0.197 | 0.64 ± 0.133 |
| Total nitrogen (mg/L)^c^ | 1.078 | 0.53 ± 0.28 | 0.44 ± 0.20 | 0.36 ± 0.15 | 0.36 ± 0.15 |

^a^For each bioreactor average and standard deviation calculation, n=11 (measured once per week)

^b^For each bioreactor average and standard deviation calculation, n = 33 (measured in triplicate each week)

^c^For each bioreactor average and standard deviation calculation, n = 9 (measured once per week, no data for weeks 9 and 10)

**Table S2. Water quality for flushed Bethlehem water (stored composite) and 9-week averages^a^**

| Water quality parameter | Stored composite water | Bioreactor | | | |
| --- | --- | --- | --- | --- | --- |
|  |  | A | B | C | D |
| Temperature (°C) | 21.1 ± 0.83 | 27.1 ± 1.21 | 26.7 ± 1.03 | 26.4 ± 0.88 | 26.2 ± 0.76 |
| pH^b^ | 8.22 ± 0.32 | 7.52 ± 0.15 | 7.55 ± 0.11 | 7.58 ± 0.12 | 7.62 ± 0.39 |
| Conductivity (µs/cm) ^b^ | 81.91 ± 6.21 | 95.6 ± 7.74 | 95.0 ± 6.28 | 99.5 ± 8.06 | 92.9 ± 3.77 |
| Turbidity (NTU) ^b^ | 547.7 ± 42.38 | 55.9 ± 25.13 | 31.9 ± 18.9 | 85.7 ± 49.6 | 121.3 ± 96.22 |
| Phosphorus (mg/L as P) (Filtered)^d^ | 0.069 ± 0.069 | 0.099 ± 0.0 | 0.11 ± 0.02 | 0.06 ± 0.0 | 0.052 ± 0.03 |
| Hardness (mg/L as CaCO_3_) | 15.97 ± 1.87 | 20.2 ± 1.53 | 21.3 ± 1.79 | 22.8 ± 2.86 | 18.8 ± 1.25 |
| Alkalinity (mg/L as CaCO_3_) | 18.3 ± 2.39 | 18.9 ± 2.06 | 18.9 ± 2.06 | 21.1 ± 3.29 | 17.5 ± 2.23 |
| TOC (mg/L as C) | 0.8784 ± 0.11 | 4.557 ± 3.81 | 4.26 ± 3.69 | 5.584 ± 3.88 | 6.147 ± 3.9 |
| Nitrate-Nitrogen (mg/L as NO_3_^-^)^c^ | 0 ± 0 | 0.175 ± 0.05 | 0.125 ± 0.05 | 0.15 ± 0.05 | 0.025 ± 0.05 |
| Ammonia-Nitrogen (mg/L as NH_3_) ^c^ | 0.345 ± 0.01 | 0.038 ± 0.01 | 0.018 ± 0.01 | 0.04 ± 0.02 | 0.088 ± 0.07 |
| Nitrite-Nitrogen (mg/L as NO_2_^-^) ^c^ | 0.01 ± 0.01 | 0.009 ± 0.00 | 0.007 ± 0.00 | 0.005 ± 0.00 | 0.015 ± 0.01 |

^a^ unless otherwise noted, n=9 for water in bioreactors and n=10 for the stored water (including the initial baseline reading) ±SD.

^b^ a single sample each week was measured in triplicate (n=27 for water in bioreactors, n=30 for the stored water)

^c^ a single sample was measured in triplicate from week 6 (n=6 for water in bioreactors and the stored water)

^d^ a single sample each week was measured in triplicate from week 8 (n=2 for water in bioreactors and the stored water)

| **Table S3. Steady-state biofilm characteristics generated by COMSTAT program** | | | | | | |
| --- | --- | --- | --- | --- | --- | --- |
|  | Mean thickness (µm) | Roughness coefficient | Bio-volume (µ3/µ2) | Surface  area  (µ2) | Surface area to volume ratio (µ2/µ3) | Maximum thickness (µm) |
| Golden State Water | 24.17  ± 7.10 | 0.51  ± 0.34 | 11.82  ± 2.71 | (1.92E+06)  ± (4.80E+05) | 1.38  ± 0.15 | 37.45  ± 4.64 |
| Bethlehem Water | 19.44  ± 10.50 | 0.65  ± 0.43 | 7.35  ± 4.11 | (2.21E+06)  ± (1.04E+06) | 2.56  ± 0.38 | 32.22  ± 9.83 |

**Table S4. Taxonomy of V6 sequences differentially represented between biofilm flushed from eight sites in the Golden State distribution system and biofilms recovered from four bioreactors.**

Columns are labeled as follows: Sample in which a particular taxon was over-represented (flushed biofilm used to seed reactors, “seed”; bioreactor biofilm, “reactor”); LDA, linear discriminant analysis value; pValue; Size in %; Taxonomy. The analysis is based on 10,000 raw sequences per sample.

Table S4 is provided as a separate file.

**Table S5. Classification of Operational Taxonomic Units and sequences indicate that *Alphaproteobacteria* expanded in bioreactors seeded with Bethlehem water**

|  | OTUs^1^ | | Sequence reads | |
| --- | --- | --- | --- | --- |
|  | Seed | Reactor | Seed | Reactor |
| *Alphaproteobacteria* | 8 | 25 | 993 | 81042 |
| Other | 96 | 6 | 10252 | 18293 |
| Total | 104 | 31 | 11245 | 99335 |

^1^Operational Taxonomic Units include sequences with an average distance of 0.01.

**Table S6. Taxonomy of V1V2 sequences differentially represented between biofilm flushed from the Bethlehem distribution system and biofilms recovered from four bioreactors.**

Columns are labeled as in Table A4. The analysis is based on 10,000 raw sequences per sample.

Table S6 is provided as a separate file.


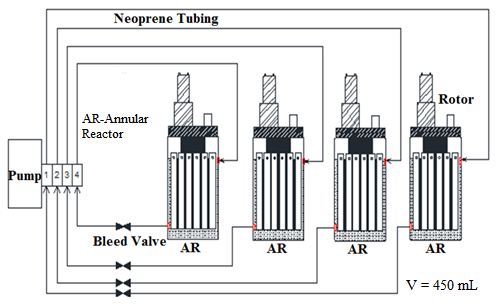


F**igure S1. Schematic diagram of the closed-loop rotating annular bioreactor system.** The pump was equipped with four pump heads shown left, each dedicated to a single bioreactor. Bleed valves were installed in each loop to allow for sampling and refreshment of water. The working volume (V) of each bioreactor was 450 mL

**Figure S2. Biofilm characteristics generated from the COMSTAT program analysis of SCLM z-stack images**. Error bars indicate one standard deviation.


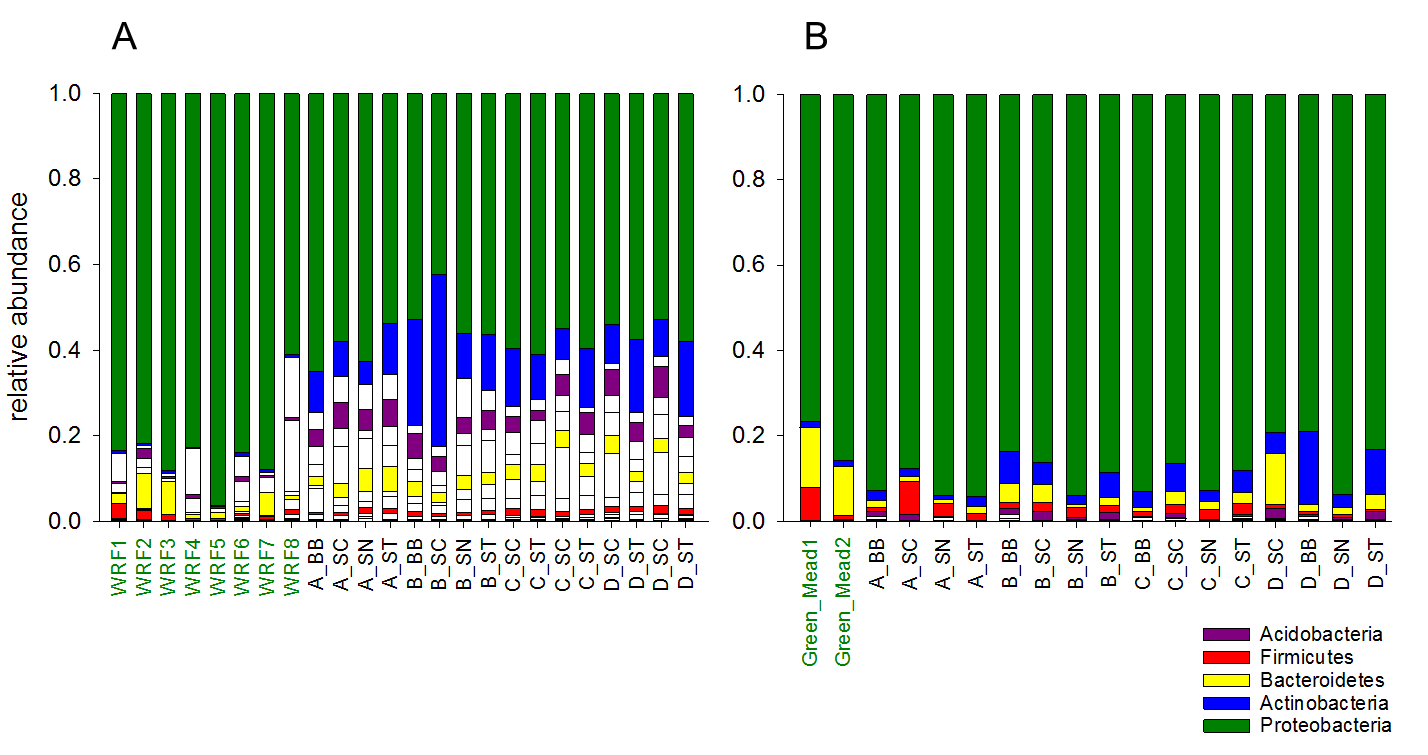


**Figure S3. Phylum-level taxonomy of bacterial populations from flushed and bioreactor biofilms.**

Bars are labelled as in Figures 2 and 4, where A, B, C and D indicate reactor and the 2-letter code following the underscore the biofilm recovery method. A, first experiment with Golden State water; B, second experiment with Bethlehem water. Green font indicates flushed samples. The most abundant phyla are color-coded as shown in the key. In order of increasing abundance, the other phyla detected in experiment 1 and not shown in the key are, Elusimicrobia BRC1, TM7, Fusobacteria, Armatimonadetes, Lentisphaerae, Chrysiogenetes, Tenericutes, WS3, Thermotogae, Thermodesulfobacteria, Fibrobacteres, Deferribacteres, OD1, Deinococcus-Thermus, Chlorobi, Caldiserica, Aquificae, Spirochaetes, Cyanobacteria_Chloroplast, Chlamydiae, Gemmatimonadetes, Chloroflexi, Synergistetes, Verrucomicrobia, Planctomycetes and Nitrospira. Bar graph shown in panel A is based on 275,530 sequences; B, 166,062 sequences.

**Supporting references**

1. Lawrence JR, Swerhone GD, Neu TR. A simple rotating annular reactor for replicated biofilm studies. J Microbiol Methods. 2000;42(3):215-24. Epub 2000/10/25. doi: S0167701200001950 [pii]. PubMed PMID: 11044565.

2. Trulear MG, Characklis WG. Dynamics of Biofilm Processes. J Water Pollut Con F. 1982;54(9):1288-301. PubMed PMID: WOS:A1982PE46600009.

3. Baker GC, Smith JJ, Cowan DA. Review and re-analysis of domain-specific 16S primers. J Microbiol Methods. 2003;55(3):541-55. Epub 2003/11/11. doi: S0167701203002276 [pii]. PubMed PMID: 14607398.

4. Hamady M, Walker JJ, Harris JK, Gold NJ, Knight R. Error-correcting barcoded primers for pyrosequencing hundreds of samples in multiplex. Nat Methods. 2008;5(3):235-7. Epub 2008/02/12. doi: nmeth.1184 [pii]

10.1038/nmeth.1184. PubMed PMID: 18264105; PubMed Central PMCID: PMC3439997.

5. Koenig JE, Spor A, Scalfone N, Fricker AD, Stombaugh J, Knight R, et al. Succession of microbial consortia in the developing infant gut microbiome. Proc Natl Acad Sci U S A. 2011;108 Suppl 1:4578-85. Epub 2010/07/30. doi: 1000081107 [pii]

10.1073/pnas.1000081107. PubMed PMID: 20668239; PubMed Central PMCID: PMC3063592.

6. Schloss PD, Westcott SL, Ryabin T, Hall JR, Hartmann M, Hollister EB, et al. Introducing mothur: open-source, platform-independent, community-supported software for describing and comparing microbial communities. Appl Environ Microbiol. 2009;75(23):7537-41. Epub 2009/10/06. doi: AEM.01541-09 [pii]

10.1128/AEM.01541-09. PubMed PMID: 19801464; PubMed Central PMCID: PMC2786419.

7. Sievers F, Wilm A, Dineen D, Gibson TJ, Karplus K, Li W, et al. Fast, scalable generation of high-quality protein multiple sequence alignments using Clustal Omega. Mol Syst Biol. 2011;7:539. doi: 10.1038/msb.2011.75. PubMed PMID: 21988835; PubMed Central PMCID: PMC3261699.

8. Edgar RC, Haas BJ, Clemente JC, Quince C, Knight R. UCHIME improves sensitivity and speed of chimera detection. Bioinformatics. 2011;27(16):2194-200. doi: 10.1093/bioinformatics/btr381. PubMed PMID: 21700674; PubMed Central PMCID: PMC3150044.

9. Lozupone C, Hamady M, Knight R. UniFrac - an online tool for comparing microbial community diversity in a phylogenetic context. BMC Bioinformatics. 2006;7:371-85. Epub 2006/08/09. doi: 1471-2105-7-371 [pii]

10.1186/1471-2105-7-371. PubMed PMID: 16893466; PubMed Central PMCID: PMC1564154.

10. Peakall R, Smouse PE. GenAlEx 6.5: genetic analysis in Excel. Population genetic software for teaching and research--an update. Bioinformatics. 2012;28(19):2537-9. Epub 2012/07/24. doi: bts460 [pii]

10.1093/bioinformatics/bts460. PubMed PMID: 22820204; PubMed Central PMCID: PMC3463245.

11. Segata N, Izard J, Waldron L, Gevers D, Miropolsky L, Garrett WS, et al. Metagenomic biomarker discovery and explanation. Genome Biol. 2011;12(6):R60. doi: 10.1186/gb-2011-12-6-r60. PubMed PMID: 21702898; PubMed Central PMCID: PMC3218848.

12. Wang Q, Garrity GM, Tiedje JM, Cole JR. Naive Bayesian classifier for rapid assignment of rRNA sequences into the new bacterial taxonomy. Appl Environ Microbiol. 2007;73(16):5261-7. Epub 2007/06/26. doi: AEM.00062-07 [pii]

10.1128/AEM.00062-07. PubMed PMID: 17586664; PubMed Central PMCID: PMC1950982.

13. Cole JR, Wang Q, Fish JA, Chai B, McGarrell DM, Sun Y, et al. Ribosomal Database Project: data and tools for high throughput rRNA analysis. Nucleic Acids Res. 2014;42(Database issue):D633-42. doi: 10.1093/nar/gkt1244. PubMed PMID: 24288368; PubMed Central PMCID: PMC3965039.

14. Gotelli NJ, Colwell RK. Quantifying biodiversity: procedures and pitfalls in the measurement and comparison of species richness. Ecology Letters. 2001;4(4):379-91. PubMed PMID: ISI:000170417800015.

15. Braak Ct, Šmilauer P. CANOCO reference manual and CanoDraw for Windows user's guide: software for canonical community ordination (version 4.5). Microcomputer Power, Ithaca, New York. 2002.

16. Legendre P, Legendre LF. Numerical ecology: Elsevier; 2012.
