# Supplementary material for: Impact of Bioreactor Environment and Recovery Method on the Profile of Bacterial Populations from Water Distribution Systems
Source: PLoS One. 2015 Jul 21;10(7):e0133427. doi: 10.1371/journal.pone.0133427 (PMC4509647; doi:10.1371/journal.pone.0133427)
Supplement: S1 Table — (PDF) [file pone.0133427.s002.pdf]

seed	LDA	pValue	Size	%	Taxonomy
seed	4.5587	3.948E-08	13145	29.029195	Bacteria(100);"Proteobacteria"(100);Gammaproteobacteria(100);Chromatiales(100);Chromatiaceae(100);Rheinheimera(100);
seed	3.8148	0.0131159	6591	14.555452	Bacteria(100);unclassified(100);unclassified(100);unclassified(100);unclassified(100);unclassified(100);
seed	4.10977	1.567E-05	4880	10.776909	Bacteria(100);"Proteobacteria"(100);Gammaproteobacteria(100);Methylococcales(100);Methylococcaceae(100);Methylomicrobium(100);
seed	4.02385	1.992E-06	4562	10.074643	Bacteria(100);"Proteobacteria"(100);Gammaproteobacteria(100);unclassified(100);unclassified(100);unclassified(100);
seed	3.8136	1.528E-05	2388	5.2736187	Bacteria(100);"Proteobacteria"(100);Alphaproteobacteria(100);Sphingomonadales(100);Erythrobacteraceae(100);Porphyrobacter(100);
seed	3.38666	4.357E-07	1540	3.4009099	Bacteria(100);"Proteobacteria"(100);Alphaproteobacteria(100);Sphingomonadales(100);Sphingomonadaceae(100);unclassified(100);
seed	3.52416	0.0318783	1312	2.8973985	Bacteria(100);"Proteobacteria"(100);Betaproteobacteria(100);Burkholderiales(100);unclassified(100);unclassified(100);
seed	2.87246	0.0051159	1279	2.8245219	Bacteria(100);"Proteobacteria"(100);unclassified(100);unclassified(100);unclassified(100);unclassified(100);
seed	3.28499	0.0021978	1141	2.519765	Bacteria(100);"Proteobacteria"(100);Betaproteobacteria(100);Burkholderiales(100);Comamonadaceae(100);Variovorax(100);
seed	3.06173	6.434E-07	1107	2.44468	Bacteria(100);"Proteobacteria"(100);Alphaproteobacteria(100);unclassified(100);unclassified(100);unclassified(100);
seed	3.11682	0.0004961	984	2.1730489	Bacteria(100);"Proteobacteria"(100);Alphaproteobacteria(100);Rhodospirillales(100);unclassified(100);unclassified(100);
seed	3.29926	0.0001935	970	2.1421315	Bacteria(100);"Proteobacteria"(100);Gammaproteobacteria(100);Gammaproteobacteria_incertae_sedis(100);Simiduia(100);
seed	2.98818	2.35E-06	778	1.718122	Bacteria(100);"Proteobacteria"(100);Alphaproteobacteria(100);Rhizobiales(100);unclassified(100);unclassified(100);
seed	3.67726	0.0002782	721	1.5922442	Bacteria(100);"Proteobacteria"(100);Gammaproteobacteria(100);Methylococcales(100);Methylococcaceae(100);unclassified(100);
seed	3.08985	0.0002782	696	1.5370346	Bacteria(100);"Bacteroidetes"(100);Flavobacteria(100);"Flavobacteriales"(100);Flavobacteriaceae(100);unclassified(100);
seed	3.13476	1.86E-07	405	0.8943951	Bacteria(100);"Proteobacteria"(100);Betaproteobacteria(100);Methylophilales(100);Methylophilaceae(100);Methylotenera(100);
seed	2.86356	3.965E-05	400	0.8833532	Bacteria(100);"Proteobacteria"(100);Betaproteobacteria(100);unclassified(100);unclassified(100);unclassified(100);
seed	3.14661	0.000153	374	0.8259353	Bacteria(100);"Proteobacteria"(100);Betaproteobacteria(100);Rhodocyclales(100);Rhodocyclaceae(100);unclassified(100);
seed	3.32028	0.0271302	348	0.7685173	Bacteria(100);"Proteobacteria"(100);Gammaproteobacteria(100);Methylococcales(100);Methylococcaceae(100);Methylobacter(100);
seed	2.97566	4.357E-07	308	0.680182	Bacteria(100);"Proteobacteria"(100);Betaproteobacteria(100);Rhodocyclales(100);Rhodocyclaceae(100);Zoogloea(100);
seed	2.80244	3.319E-05	186	0.4107592	Bacteria(100);"Planctomycetes"(100);"Planctomycetacia"(100);Planctomycetales(100);Planctomycetaceae(100);unclassified(100);
seed	2.61638	8.806E-06	151	0.3334658	Bacteria(100);"Proteobacteria"(100);Alphaproteobacteria(100);Caulobacterales(100);Caulobacteraceae(100);Caulobacter(100);
seed	2.62429	0.0029335	134	0.2959233	Bacteria(100);"Proteobacteria"(100);Alphaproteobacteria(100);Rhizobiales(100);Methylocystaceae(100);Methylocystis(100);
seed	2.61155	4.357E-07	123	0.2716311	Bacteria(100);"Proteobacteria"(100);Alphaproteobacteria(100);Rhizobiales(100);Rhodobiaceae(100);Parvibaculum(100);
seed	2.74829	0.0001117	101	0.2230467	Bacteria(100);"Proteobacteria"(100);Alphaproteobacteria(100);Rhodospirillales(100);Acetobacteraceae(100);Stella(100);
seed	2.606	0.0116421	88	0.1943377	Bacteria(100);"Proteobacteria"(100);Alphaproteobacteria(100);Sneathiellales(100);Sneathiellaceae(100);Sneathiella(100);
seed	2.61303	2.547E-05	84	0.1855042	Bacteria(100);"Proteobacteria"(100);Betaproteobacteria(100);Rhodocyclales(100);Rhodocyclaceae(100);Azospira(100);
seed	2.34743	0.0046198	72	0.1590036	Bacteria(100);"Proteobacteria"(100);Alphaproteobacteria(100);Caulobacterales(100);Caulobacteraceae(100);unclassified(100);
seed	2.42213	0.0084428	50	0.1104192	Bacteria(100);"Proteobacteria"(100);Alphaproteobacteria(100);Sphingomonadales(100);Sphingomonadaceae(100);Sphingosinicella(100);
seed	2.26157	0.0005773	49	0.1082108	Bacteria(100);"Proteobacteria"(100);Gammaproteobacteria(100);"Enterobacteriales"(100);Enterobacteriaceae(100);Escherichia/Shigella(100);
seed	2.23682	0.0002782	47	0.103794	Bacteria(100);"Proteobacteria"(100);Betaproteobacteria(100);Burkholderiales(100);Comamonadaceae(100);unclassified(100);
seed	2.35996	0.0002782	37	0.0817102	Bacteria(100);"Proteobacteria"(100);Deltaproteobacteria(100);unclassified(100);unclassified(100);unclassified(100);
seed	2.37528	4.357E-07	32	0.0706683	Bacteria(100);"Planctomycetes"(100);"Planctomycetacia"(100);Planctomycetales(100);Planctomycetaceae(100);Singulisphaera(100);
seed	2.25365	0.0002388	28	0.0618347	Bacteria(100);"Proteobacteria"(100);Betaproteobacteria(100);Burkholderiales(100);Burkholderiaceae(100);Ralstonia(100);
seed	2.12107	0.0038569	27	0.0596263	Bacteria(100);"Proteobacteria"(100);Betaproteobacteria(100);Burkholderiales(100);Comamonadaceae(100);Simplicispira(100);
seed	2.07615	0.030355	22	0.0485844	Bacteria(100);"Proteobacteria"(100);Alphaproteobacteria(100);Rhodobacterales(100);Rhodobacteraceae(100);Rhodobacter(100);
seed	2.01695	0.0020858	20	0.0441677	Bacteria(100);"Proteobacteria"(100);Alphaproteobacteria(100);Rhizobiales(100);Hyphomicrobiaceae(100);Devosia(100);
seed	2.11748	3.614E-05	20	0.0441677	Bacteria(100);"Proteobacteria"(100);Betaproteobacteria(100);Burkholderiales(100);Comamonadaceae(100);Pelomonas(100);
seed	2.15878	3.614E-05	18	0.0397509	Bacteria(100);"Proteobacteria"(100);Alphaproteobacteria(100);Rhodospirillales(100);Rhodospirillaceae(100);unclassified(100);
seed	2.04391	0.0002782	16	0.0353341	Bacteria(100);"Proteobacteria"(100);Alphaproteobacteria(100);Sphingomonadales(100);Sphingomonadaceae(100);Novosphingobium(100);
seed	2.07002	3.614E-05	15	0.0331257	Bacteria(100);"Firmicutes"(100);"Clostridia"(100);Clostridiales(100);"Lachnospiraceae"(100);unclassified(100);
seed	2.06245	3.614E-05	14	0.0309174	Bacteria(100);"Proteobacteria"(100);Alphaproteobacteria(100);Rhodobacterales(100);Rhodobacteraceae(100);unclassified(100);

seed	2.03396	4.214E-06	14	0.0309174	Bacteria(100);"Proteobacteria"(100);Alphaproteobacteria(100);unclassified(100);unclassified(100);unclassified(100);
seed	2.03358	3.614E-05	5	0.0110419	Bacteria(100);"Proteobacteria"(100);Deltaproteobacteria(100);Bdellovibrionales(100);Bacteriovoracaceae(100);Peredibacter(100);

reactor	LDA	pValue	OTU	Size	%	Taxonomy
reactor	3.49074	0.0003589	Otu00012	39459	28.16046	Bacteria(100);unclassified(100);unclassified(100);unclassified(100);unclassified(100);unclassified(100);
reactor	4.09751	2.549E-05	Otu00002	10183	7.2672385	Bacteria(100);"Proteobacteria"(100);Gammaproteobacteria(100);Pseudomonadales(100);Pseudomonadaceae(100);Azotobacter(100);
reactor	3.61811	2.893E-05	Otu00013	8592	6.1317994	Bacteria(100);"Proteobacteria"(100);unclassified(100);unclassified(100);unclassified(100);unclassified(100);
reactor	4.0165	2.549E-05	Otu00003	7736	5.5209032	Bacteria(100);"Proteobacteria"(100);Alphaproteobacteria(100);Rhodospirillales(100);unclassified(100);unclassified(100);
reactor	4.01079	2.549E-05	Otu00004	7292	5.2040365	Bacteria(100);"Actinobacteria"(100);Actinobacteria(100);Actinomycetales(100);unclassified(100);unclassified(100);
reactor	3.60084	2.675E-05	Otu00019	6168	4.4018784	Bacteria(100);"Actinobacteria"(100);Actinobacteria(100);Actinomycetales(100);Mycobacteriaceae(100);Mycobacterium(100);
reactor	3.51851	2.549E-05	Otu00024	5303	3.7845592	Bacteria(100);"Proteobacteria"(100);Alphaproteobacteria(100);unclassified(100);unclassified(100);unclassified(100);
reactor	3.23732	0.0046334	Otu00063	3682	2.6277101	Bacteria(100);"Proteobacteria"(100);Betaproteobacteria(100);Burkholderiales(100);unclassified(100);unclassified(100);
reactor	3.65125	2.549E-05	Otu00014	3551	2.5342202	Bacteria(100);"Proteobacteria"(100);Betaproteobacteria(100);Burkholderiales(100);Burkholderiaceae(100);Limnobacter(100);
reactor	2.12328	0.0274426	Otu00660	3050	2.1766746	Bacteria(100);"Acidobacteria"(100);Acidobacteria_Gp10(100);unclassified(100);unclassified(100);unclassified(100);
reactor	3.28681	2.675E-05	Otu00048	2718	1.9397382	Bacteria(100);"Proteobacteria"(100);Gammaproteobacteria(100);unclassified(100);unclassified(100);unclassified(100);
reactor	3.5464	2.549E-05	Otu00022	2238	1.5971796	Bacteria(100);"Chloroflexi"(100);Caldilineae(100);Caldilineales(100);Caldilineaceae(100);Caldilinea(100);
reactor	2.96832	5.153E-05	Otu00122	2000	1.4273276	Bacteria(100);"Actinobacteria"(100);Actinobacteria(100);unclassified(100);unclassified(100);unclassified(100);
reactor	3.3785	2.549E-05	Otu00033	1946	1.3887898	Bacteria(100);"Proteobacteria"(100);Alphaproteobacteria(100);Rhizobiales(100);Hyphomicrobiaceae(100);Pedomicrobium(100);
reactor	3.20937	2.549E-05	Otu00058	1740	1.241775	Bacteria(100);"Proteobacteria"(100);Alphaproteobacteria(100);Rhodospirillales(100);Acetobacteraceae(100);Roseomonas(100);
reactor	3.04507	0.0003482	Otu00101	1692	1.2075192	Bacteria(100);"Proteobacteria"(100);Betaproteobacteria(100);unclassified(100);unclassified(100);unclassified(100);
reactor	3.14161	0.0001007	Otu00077	1688	1.2046645	Bacteria(100);"Proteobacteria"(100);Gammaproteobacteria(100);Xanthomonadales(100);Xanthomonadaceae(100);unclassified(100);
reactor	3.32402	2.675E-05	Otu00045	1676	1.1961005	Bacteria(100);"Proteobacteria"(100);Betaproteobacteria(100);Burkholderiales(100);Comamonadaceae(100);Delftia(100);
reactor	2.69919	0.0030292	Otu00114	1618	1.154708	Bacteria(100);"Proteobacteria"(100);Alphaproteobacteria(100);Rhizobiales(100);Hyphomicrobiaceae(100);Hyphomicrobium(100);
reactor	3.5894	2.549E-05	Otu00021	1555	1.1097472	Bacteria(100);"Proteobacteria"(100);Gammaproteobacteria(100);Xanthomonadales(100);Xanthomonadaceae(100);Rhodanobacter(100);
reactor	3.25893	2.549E-05	Otu00051	1468	1.0476585	Bacteria(100);"Proteobacteria"(100);Gammaproteobacteria(100);"Enterobacteriales"(100);Enterobacteriaceae(100);Erwinia(100);
reactor	3.24613	5.153E-05	Otu00055	1340	0.9563095	Bacteria(100);"Proteobacteria"(100);Betaproteobacteria(100);Burkholderiales(100);Comamonadaceae(100);unclassified(100);
reactor	2.90548	2.549E-05	Otu00138	1283	0.9156307	Bacteria(100);"Verrucomicrobia"(100);unclassified(100);unclassified(100);unclassified(100);unclassified(100);
reactor	3.52472	5.153E-05	Otu00026	1280	0.9134897	Bacteria(100);"Actinobacteria"(100);Actinobacteria(100);Actinomycetales(100);Nocardiaceae(100);Nocardia(100);
reactor	3.12186	5.153E-05	Otu00082	1091	0.7786072	Bacteria(100);"Actinobacteria"(100);Actinobacteria(100);Actinomycetales(100);Nocardioidaceae(100);Aeromicrobium(100);
reactor	3.05862	2.549E-05	Otu00094	1091	0.7786072	Bacteria(100);"Chloroflexi"(100);unclassified(100);unclassified(100);unclassified(100);unclassified(100);
reactor	3.04182	4.298E-05	Otu00084	1021	0.7286507	Bacteria(100);"Proteobacteria"(100);Alphaproteobacteria(100);Rhizobiales(100);Bradyrhizobiaceae(100);Bradyrhizobium(100);
reactor	3.28194	0.0003482	Otu00050	1019	0.7272234	Bacteria(100);"Verrucomicrobia"(100);Verrucomicrobiae(100);Verrucomicrobiales(100);Verrucomicrobiaceae(100);unclassified(100);
reactor	3.43144	2.549E-05	Otu00032	1017	0.7257961	Bacteria(100);"Bacteroidetes"(100);unclassified(100);unclassified(100);unclassified(100);unclassified(100);
reactor	3.15522	3.243E-05	Otu00073	995	0.7100955	Bacteria(100);"Proteobacteria"(100);Gammaproteobacteria(100);Aeromonadales(100);Aeromonadaceae(100);Aeromonas(100);
reactor	2.96485	2.549E-05	Otu00121	975	0.6958222	Bacteria(100);"Proteobacteria"(100);Betaproteobacteria(100);Burkholderiales(100);Burkholderiales_incertae_sedis(100);unclassified(100);
reactor	2.69162	4.9E-05	Otu00226	940	0.670844	Bacteria(100);"Proteobacteria"(100);Alphaproteobacteria(100);Rhizobiales(100);Hyphomicrobiaceae(100);unclassified(100);
reactor	3.02341	5.994E-05	Otu00102	940	0.670844	Bacteria(100);"Proteobacteria"(100);Gammaproteobacteria(100);Xanthomonadales(100);Xanthomonadaceae(100);Luteimonas(100);
reactor	2.41212	0.0017842	Otu00443	906	0.6465794	Bacteria(100);"Proteobacteria"(100);Alphaproteobacteria(100);Rhodospirillales(100);Acetobacteraceae(100);unclassified(100);
reactor	3.0619	3.923E-05	Otu00088	849	0.6059006	Bacteria(100);"Actinobacteria"(100);Actinobacteria(100);Actinomycetales(100);Microbacteriaceae(100);Plantibacter(100);
reactor	2.97444	0.0001007	Otu00111	839	0.5987639	Bacteria(100);"Actinobacteria"(100);Actinobacteria(100);Acidimicrobiales(100);unclassified(100);unclassified(100);
reactor	3.28522	5.153E-05	Otu00049	767	0.5473801	Bacteria(100);"Proteobacteria"(100);Gammaproteobacteria(100);Thiotrichales(100);unclassified(100);unclassified(100);
reactor	2.87188	0.0001007	Otu00157	748	0.5338205	Bacteria(100);"Proteobacteria"(100);Betaproteobacteria(100);Burkholderiales(100);Comamonadaceae(100);Schlegelella(100);
reactor	2.89998	0.0003646	Otu00141	665	0.4745864	Bacteria(100);"Proteobacteria"(100);Alphaproteobacteria(100);Rhodospirillales(100);Acetobacteraceae(100);Rhodopila(100);
reactor	2.66662	0.0001902	Otu00250	655	0.4674498	Bacteria(100);"Actinobacteria"(100);Actinobacteria(100);Acidimicrobidae_incertae_sedis(100);Ilumatobacter(100);
reactor	2.91	2.549E-05	Otu00146	627	0.4474672	Bacteria(100);"Actinobacteria"(100);Actinobacteria(100);Actinomycetales(100);Nocardiaceae(100);Gordonia(100);
reactor	2.86039	2.549E-05	Otu00165	562	0.4010791	Bacteria(100);"Proteobacteria"(100);Betaproteobacteria(100);Rhodocyclales(100);Rhodocyclaceae(100);Methyloversatilis(100);

reactor	2.8458	2.549E-05	Otu00171	498	0.3554046	Bacteria(100);"Proteobacteria"(100);Alphaproteobacteria(100);Sphingomonadales(100);Sphingomonadaceae(100);Blastomonas(100);
reactor	3.0577	2.549E-05	Otu00099	480	0.3425586	Bacteria(100);"Bacteroidetes"(100);"Sphingobacteria"(100);"Sphingobacteriales"(100);unclassified(100);unclassified(100);
reactor	2.59064	0.0001902	Otu00298	479	0.341845	Bacteria(100);"Verrucomicrobia"(100);Opitutae(100);Opitutales(100);Opitutaceae(100);Alterococcus(100);
reactor	2.75484	0.010947	Otu00216	470	0.335422	Bacteria(100);"Actinobacteria"(100);Actinobacteria(100);Actinomycetales(100);Microbacteriaceae(100);unclassified(100);
reactor	2.53358	0.0002665	Otu00342	424	0.3025935	Bacteria(100);"Verrucomicrobia"(100);Subdivision3(100);unclassified(100);unclassified(100);unclassified(100);
reactor	2.72069	0.0002597	Otu00222	374	0.2669103	Bacteria(100);"Proteobacteria"(100);Gammaproteobacteria(100);Xanthomonadales(100);Xanthomonadaceae(100);Xanthomonas(100);
reactor	2.818	0.0046334	Otu00175	263	0.1876936	Bacteria(100);"Proteobacteria"(100);Alphaproteobacteria(100);Caulobacterales(100);Caulobacteraceae(100);unclassified(100);
reactor	2.60767	0.0046334	Otu00272	221	0.1577197	Bacteria(100);"Proteobacteria"(100);Gammaproteobacteria(100);Legionellales(100);Coxiellaceae(100);Aquicella(100);
reactor	2.45369	0.0003482	Otu00396	218	0.1555787	Bacteria(100);"Proteobacteria"(100);Betaproteobacteria(100);Burkholderiales(100);Burkholderiales_incertae_sedis(100);Aquabacterium(100)
reactor	2.22626	0.0072014	Otu00641	162	0.1156135	Bacteria(100);"Proteobacteria"(100);Alphaproteobacteria(100);Rhizobiales(100);unclassified(100);unclassified(100);
reactor	2.03193	0.014829	Otu00527	150	0.1070496	Bacteria(100);"Chloroflexi"(100);Anaerolineae(100);Anaerolineales(100);Anaerolineaceae(100);unclassified(100);
reactor	2.18188	0.0106999	Otu00331	134	0.095631	Bacteria(100);"Planctomycetes"(100);"Planctomycetacia"(100);Planctomycetales(100);Planctomycetaceae(100);Schlesneria(100);
reactor	2.00542	0.0407404	Otu00337	130	0.0927763	Bacteria(100);"Proteobacteria"(100);Alphaproteobacteria(100);Rhodobacterales(100);Rhodobacteraceae(100);unclassified(100);
reactor	2.17059	0.0072014	Otu00706	130	0.0927763	Bacteria(100);"Proteobacteria"(100);Alphaproteobacteria(100);Sphingomonadales(100);Sphingomonadaceae(100);Sphingobium(100);
reactor	2.49304	5.994E-05	Otu00344	126	0.0899216	Bacteria(100);"Proteobacteria"(100);Alphaproteobacteria(100);Caulobacterales(100);Hyphomonadaceae(100);Hyphomonas(100);
reactor	2.42878	0.0018816	Otu00369	117	0.0834987	Bacteria(100);"Proteobacteria"(100);Betaproteobacteria(100);Burkholderiales(100);Comamonadaceae(100);Acidovorax(100);
reactor	2.45754	0.0162998	Otu00374	116	0.082785	Bacteria(100);"Proteobacteria"(100);Alphaproteobacteria(100);Caulobacterales(100);Caulobacteraceae(100);Caulobacter(100);
reactor	2.21967	5.153E-05	Otu00639	99	0.0706527	Bacteria(100);"Acidobacteria"(100);unclassified(100);unclassified(100);unclassified(100);unclassified(100);
reactor	2.29378	0.0016098	Otu00517	85	0.0606614	Bacteria(100);"Planctomycetes"(100);"Planctomycetacia"(100);Planctomycetales(100);Planctomycetaceae(100);unclassified(100);
reactor	2.12597	0.0382458	Otu00615	67	0.0478155	Bacteria(100);"Proteobacteria"(100);Betaproteobacteria(100);Burkholderiales(100);Comamonadaceae(100);Diaphorobacter(100);
reactor	2.16051	0.000489	Otu00626	65	0.0463881	Bacteria(100);"Proteobacteria"(100);Alphaproteobacteria(100);Caulobacterales(100);Caulobacteraceae(100);Phenylobacterium(100);
reactor	2.19072	0.0003482	Otu00670	60	0.0428198	Bacteria(100);"Actinobacteria"(100);Actinobacteria(100);Solirubrobacterales(100);Conexibacteraceae(100);Conexibacter(100);
reactor	2.19256	0.010947	Otu00666	60	0.0428198	Bacteria(100);"Bacteroidetes"(100);"Sphingobacteria"(100);"Sphingobacteriales"(100);"Saprospiraceae"(100);Haliscomenobacter(100);
reactor	2.15937	0.0001902	Otu00727	53	0.0378242	Bacteria(100);"Proteobacteria"(100);Alphaproteobacteria(100);Rhizobiales(100);Phyllobacteriaceae(100);Mesorhizobium(100);
reactor	2.05357	0.003171	Otu00788	46	0.0328285	Bacteria(100);"Proteobacteria"(100);Gammaproteobacteria(100);Chromatiales(100);Ectothiorhodospiraceae(100);unclassified(100);
reactor	2.11354	0.0001007	Otu00805	44	0.0314012	Bacteria(100);"Proteobacteria"(100);Betaproteobacteria(100);Rhodocyclales(100);Rhodocyclaceae(100);Zoogloea(100);
reactor	2.08805	0.010947	Otu00804	44	0.0314012	Bacteria(100);"Proteobacteria"(100);Gammaproteobacteria(100);Legionellales(100);Coxiellaceae(100);Aquicella(100);
reactor	2.10488	0.0072014	Otu00843	42	0.0299739	Bacteria(100);"Proteobacteria"(100);Betaproteobacteria(100);Neisseriales(100);Neisseriaceae(100);unclassified(100);
