# Supplementary material for: Impact of Bioreactor Environment and Recovery Method on the Profile of Bacterial Populations from Water Distribution Systems
Source: PLoS One. 2015 Jul 21;10(7):e0133427. doi: 10.1371/journal.pone.0133427 (PMC4509647; doi:10.1371/journal.pone.0133427)
Supplement: S2 Table — (PDF) [file pone.0133427.s003.pdf]

seed	LDA	pValue	Size	%	Taxonomy
seed	4.58315	0.024589	1553	13.81058	Bacteria(100);"Proteobacteria"(100);Gammaproteobacteria(100);"Enterobacteriales"(100);Enterobacteriaceae(100);Escherichia_Shigella(71);
seed	4.53665	0.024589	1239	11.01823	Bacteria(100);"Bacteroidetes"(100);Flavobacteria(100);"Flavobacteriales"(100);Flavobacteriaceae(100);Cloacibacterium(100);
seed	4.46486	0.024589	1121	9.968875	Bacteria(100);"Proteobacteria"(100);Gammaproteobacteria(100);"Enterobacteriales"(100);Enterobacteriaceae(100);Escherichia_Shigella(100);
seed	4.27265	0.024589	1016	9.035127	Bacteria(100);"Proteobacteria"(100);Betaproteobacteria(100);Burkholderiales(100);Comamonadaceae(100);Diaphorobacter(100);
seed	4.33929	0.024293	1009	8.972877	Bacteria(100);"Proteobacteria"(100);Gammaproteobacteria(100);Legionellales(100);Legionellaceae(100);Legionella(100);
seed	4.32082	0.02745	962	8.554913	Bacteria(100);"Proteobacteria"(100);Alphaproteobacteria(100);Rhizobiales(100);Bradyrhizobiaceae(100);Bosea(100);
seed	4.40513	0.010567	771	6.856381	Bacteria(100);"Proteobacteria"(100);Betaproteobacteria(100);Burkholderiales(100);Comamonadaceae(100);Acidovorax(100);
seed	4.01551	0.024293	567	5.042241	Bacteria(100);"Proteobacteria"(100);Gammaproteobacteria(100);Legionellales(100);Legionellaceae(100);Legionella(100);
seed	3.86005	0.024515	540	4.802134	Bacteria(100);"Proteobacteria"(100);Gammaproteobacteria(100);Legionellales(100);Legionellaceae(100);Legionella(100);
seed	4.07939	0.024293	478	4.250778	Bacteria(100);"Bacteroidetes"(100);Flavobacteria(100);"Flavobacteriales"(100);Flavobacteriaceae(100);Cloacibacterium(100);
seed	4.05479	0.01867	416	3.699422	Bacteria(100);"Proteobacteria"(100);Betaproteobacteria(100);Burkholderiales(100);Comamonadaceae(100);Acidovorax(100);
seed	3.65823	0.004678	170	1.511783	Bacteria(100);"Proteobacteria"(100);Betaproteobacteria(100);Rhodocyclales(100);Rhodocyclaceae(100);Sulfuritalea(100);
seed	3.51767	0.004678	123	1.093819	Bacteria(100);"Proteobacteria"(100);Betaproteobacteria(100);Rhodocyclales(100);Rhodocyclaceae(100);Zoogloea(94);
seed	3.48086	0.004678	113	1.004891	Bacteria(100);"Actinobacteria"(100);Actinobacteria(100);Actinomycetales(100);Intrasporangiaceae(97);Ornithinicoccus(97);
seed	3.67726	0.004678	106	0.942641	Bacteria(100);Firmicutes(100);Clostridia(100);Clostridiales(100);Ruminococcaceae(100);Ethanoligenens(100);
seed	3.44064	0.004678	103	0.915963	Bacteria(100);"Bacteroidetes"(100);"Bacteroidia"(100);"Bacteroidales"(100);"Porphyromonadaceae"(100);Parabacteroides(100);
seed	3.59149	0.004678	87	0.773677	Bacteria(100);"Proteobacteria"(100);Gammaproteobacteria(100);"Enterobacteriales"(100);Enterobacteriaceae(100);Escherichia_Shigella(100);
seed	3.42714	0.010567	85	0.755892	Bacteria(100);"Proteobacteria"(100);Betaproteobacteria(100);Burkholderiales(100);Comamonadaceae(100);Comamonas(100);
seed	3.33087	0.004678	80	0.711427	Bacteria(100);Firmicutes(100);Negativicutes(100);Selenomonadales(100);Veillonellaceae(100);Dialister(100);
seed	3.20598	0.004678	60	0.53357	Bacteria(100);"Proteobacteria"(100);Betaproteobacteria(100);Rhodocyclales(100);Rhodocyclaceae(100);Zoogloea(100);
seed	3.19146	0.004678	58	0.515785	Bacteria(100);"Proteobacteria"(100);Betaproteobacteria(100);Rhodocyclales(100);Rhodocyclaceae(100);Dechloromonas(100);
seed	3.40022	0.004678	56	0.497999	Bacteria(100);"Proteobacteria"(100);Betaproteobacteria(100);Burkholderiales(100);Oxalobacteraceae(100);Massilia(100);
seed	3.38453	0.004678	54	0.480213	Bacteria(100);"Proteobacteria"(100);Gammaproteobacteria(100);Pseudomonadales(100);Moraxellaceae(100);Acinetobacter(100);
seed	3.31488	0.004678	46	0.409071	Bacteria(100);"Proteobacteria"(100);Betaproteobacteria(100);Burkholderiales(100);Sutterellaceae(100);Sutterella(100);
seed	3.19103	3.86E-05	28	0.249	Bacteria(100);"Proteobacteria"(100);Gammaproteobacteria(100);"Enterobacteriales"(100);Enterobacteriaceae(100);Escherichia_Shigella(72);
seed	2.99474	0.004678	22	0.195643	Bacteria(100);"Proteobacteria"(100);Betaproteobacteria(100);Burkholderiales(100);Comamonadaceae(100);Xenophilus(100);
seed	2.69832	0.020813	20	0.177857	Bacteria(100);"Proteobacteria"(100);Gammaproteobacteria(100);"Enterobacteriales"(100);Enterobacteriaceae(100);Citrobacter(100);
seed	2.7441	3.86E-05	18	0.160071	Bacteria(100);"Proteobacteria"(100);Gammaproteobacteria(100);Legionellales(100);Legionellaceae(100);Legionella(100);
seed	2.66741	3.86E-05	16	0.142285	Bacteria(100);"Proteobacteria"(100);Betaproteobacteria(100);Burkholderiales(100);Comamonadaceae(100);Acidovorax(100);
seed	2.54341	0.004678	13	0.115607	Bacteria(100);"Proteobacteria"(100);Gammaproteobacteria(100);"Enterobacteriales"(100);Enterobacteriaceae(100);Plesiomonas(100);
seed	2.38363	0.004678	9	0.080036	Bacteria(100);"Proteobacteria"(100);Betaproteobacteria(100);Rhodocyclales(100);Rhodocyclaceae(100);Dechloromonas(100);
seed	2.48217	0.00054	9	0.080036	Bacteria(100);"Proteobacteria"(100);Gammaproteobacteria(100);"Enterobacteriales"(100);Enterobacteriaceae(100);Escherichia_Shigella(100);
seed	2.33114	0.004678	8	0.071143	Bacteria(100);"Proteobacteria"(100);Gammaproteobacteria(100);"Enterobacteriales"(100);Enterobacteriaceae(100);Enterobacter(100);
seed	2.33296	0.00054	7	0.06225	Bacteria(100);"Proteobacteria"(100);Gammaproteobacteria(100);"Enterobacteriales"(100);Enterobacteriaceae(100);Escherichia_Shigella(100);
seed	2.3149	3.86E-05	7	0.06225	Bacteria(100);"Proteobacteria"(100);Gammaproteobacteria(100);"Enterobacteriales"(100);Enterobacteriaceae(100);Escherichia_Shigella(100);
seed	2.31397	3.86E-05	7	0.06225	Bacteria(100);"Proteobacteria"(100);Gammaproteobacteria(100);Legionellales(100);Legionellaceae(100);Legionella(100);
seed	2.15291	0.003811	7	0.06225	Bacteria(100);"Proteobacteria"(100);Betaproteobacteria(100);Burkholderiales(100);Comamonadaceae(100);Diaphorobacter(100);
seed	2.07602	0.020813	7	0.06225	Bacteria(100);"Proteobacteria"(100);Gammaproteobacteria(100);Legionellales(100);Legionellaceae(100);Legionella(100);
seed	2.10404	0.002027	6	0.053357	Bacteria(100);"Proteobacteria"(100);Alphaproteobacteria(100);Rhizobiales(100);Methylocystaceae(100);Methylocystis(100);
seed	2.43153	0.004678	6	0.053357	Bacteria(100);"Bacteroidetes"(100);Flavobacteria(100);"Flavobacteriales"(100);Flavobacteriaceae(100);Cloacibacterium(100);
seed	2.20885	0.004678	6	0.053357	Bacteria(100);"Proteobacteria"(100);Alphaproteobacteria(100);Rhizobiales(100);Bradyrhizobiaceae(100);Bosea(100);
seed	2.31306	0.00118	6	0.053357	Bacteria(100);"Proteobacteria"(100);Gammaproteobacteria(100);"Enterobacteriales"(100);Enterobacteriaceae(100);Salmonella(100);
seed	2.25438	3.86E-05	6	0.053357	Bacteria(100);"Proteobacteria"(100);Gammaproteobacteria(100);"Enterobacteriales"(100);Enterobacteriaceae(100);Salmonella(84);
seed	2.05411	0.004445	6	0.053357	Bacteria(100);"Proteobacteria"(100);Gammaproteobacteria(100);"Enterobacteriales"(100);Enterobacteriaceae(100);Escherichia_Shigella(100);
seed	2.1636	0.002027	6	0.053357	Bacteria(100);"Proteobacteria"(100);Gammaproteobacteria(100);Legionellales(100);Legionellaceae(100);Legionella(100);
seed	2.16993	0.00054	5	0.044464	Bacteria(100);"Proteobacteria"(100);Gammaproteobacteria(100);"Enterobacteriales"(100);Enterobacteriaceae(100);Escherichia_Shigella(100);
seed	2.13176	0.004678	5	0.044464	Bacteria(100);"Proteobacteria"(100);Gammaproteobacteria(100);"Enterobacteriales"(100);Enterobacteriaceae(100);Enterobacter(100);
seed	2.35317	0.004678	5	0.044464	Bacteria(100);"Proteobacteria"(100);Gammaproteobacteria(100);Pseudomonadales(100);Moraxellaceae(100);Acinetobacter(100);
seed	2.16129	0.00054	5	0.044464	Bacteria(100);"Proteobacteria"(100);Gammaproteobacteria(100);"Enterobacteriales"(100);Enterobacteriaceae(100);Escherichia_Shigella(100);
seed	2.20091	0.00054	5	0.044464	Bacteria(100);"Bacteroidetes"(100);Flavobacteria(100);"Flavobacteriales"(100);Flavobacteriaceae(100);Cloacibacterium(100);
seed	2.10118	3.86E-05	4	0.035571	Bacteria(100);"Proteobacteria"(100);Betaproteobacteria(100);Burkholderiales(100);Comamonadaceae(100);Acidovorax(100);
seed	2.03385	0.004678	4	0.035571	Bacteria(100);"Bacteroidetes"(100);Flavobacteria(100);"Flavobacteriales"(100);Flavobacteriaceae(100);Cloacibacterium(100);
seed	2.10045	3.86E-05	4	0.035571	Bacteria(100);"Proteobacteria"(100);Gammaproteobacteria(100);"Enterobacteriales"(100);Enterobacteriaceae(100);Salmonella(100);
seed	2.16001	3.86E-05	4	0.035571	Bacteria(100);"Bacteroidetes"(100);Flavobacteria(100);"Flavobacteriales"(100);Flavobacteriaceae(100);Cloacibacterium(100);
seed	2.25549	0.004678	4	0.035571	Bacteria(100);"Bacteroidetes"(100);Flavobacteria(100);"Flavobacteriales"(100);Flavobacteriaceae(100);Cloacibacterium(100);
seed	2.2572	0.004678	4	0.035571	Bacteria(100);"Proteobacteria"(100);Alphaproteobacteria(100);Rhizobiales(100);Methylocystaceae(100);Methylocystis(100);

seed	2.25659	0.004678	4	0.035571	Bacteria(100);"Proteobacteria"(100);Gammaproteobacteria(100);"Enterobacteriales"(100);Enterobacteriaceae(100);Escherichia_Shigella(100);
seed	2.06392	0.00118	4	0.035571	Bacteria(100);"Proteobacteria"(100);Gammaproteobacteria(100);"Enterobacteriales"(100);Enterobacteriaceae(100);unclassified;
seed	2.21037	3.86E-05	4	0.035571	Bacteria(100);"Proteobacteria"(100);Alphaproteobacteria(100);Rhizobiales(100);Bradyrhizobiaceae(100);Bradyrhizobium(75);
seed	2.09974	3.86E-05	4	0.035571	Bacteria(100);"Proteobacteria"(100);Gammaproteobacteria(100);"Enterobacteriales"(100);Enterobacteriaceae(100);Escherichia_Shigella(100);
seed	2.03531	0.004678	4	0.035571	Bacteria(100);"Bacteroidetes"(100);Flavobacteria(100);"Flavobacteriales"(100);Flavobacteriaceae(100);Cloacibacterium(100);
seed	2.06473	0.00118	4	0.035571	Bacteria(100);"Bacteroidetes"(100);Flavobacteria(100);"Flavobacteriales"(100);Flavobacteriaceae(100);Cloacibacterium(100);
seed	2.06659	0.00118	4	0.035571	Bacteria(100);"Proteobacteria"(100);Gammaproteobacteria(100);"Enterobacteriales"(100);Enterobacteriaceae(100);Salmonella(100);
seed	2.19815	0.00054	4	0.035571	Bacteria(100);"Bacteroidetes"(100);Flavobacteria(100);"Flavobacteriales"(100);Flavobacteriaceae(100);Cloacibacterium(100);
seed	2.03218	0.004678	4	0.035571	Bacteria(100);"Proteobacteria"(100);Gammaproteobacteria(100);Legionellales(100);Legionellaceae(100);Legionella(100);
seed	2.03357	0.004678	4	0.035571	Bacteria(100);"Proteobacteria"(100);Alphaproteobacteria(100);Rhizobiales(100);Bradyrhizobiaceae(100);Bosea(100);
seed	2.20918	3.86E-05	4	0.035571	Bacteria(100);"Bacteroidetes"(100);Flavobacteria(100);"Flavobacteriales"(100);Flavobacteriaceae(100);Cloacibacterium(100);
seed	2.21055	3.86E-05	4	0.035571	Bacteria(100);"Proteobacteria"(100);Gammaproteobacteria(100);"Enterobacteriales"(100);Enterobacteriaceae(100);Salmonella(75);
seed	2.25509	0.004678	4	0.035571	Bacteria(100);"Proteobacteria"(100);Alphaproteobacteria(100);Rhizobiales(100);Xanthobacteraceae(100);Pseudolabrys(100);
seed	2.05519	0.00054	4	0.035571	Bacteria(100);"Proteobacteria"(100);Gammaproteobacteria(100);"Enterobacteriales"(100);Enterobacteriaceae(100);Salmonella(100);
seed	2.25539	0.004678	4	0.035571	Bacteria(100);"Proteobacteria"(100);Gammaproteobacteria(100);"Enterobacteriales"(100);Enterobacteriaceae(100);Salmonella(100);
seed	2.08317	0.00054	4	0.035571	Bacteria(100);"Proteobacteria"(100);Gammaproteobacteria(100);"Enterobacteriales"(100);Enterobacteriaceae(100);Escherichia_Shigella(100);
seed	2.10171	3.86E-05	4	0.035571	Bacteria(100);"Proteobacteria"(100);Gammaproteobacteria(100);Legionellales(100);Legionellaceae(100);Legionella(100);
seed	2.00748	0.006923	4	0.035571	Bacteria(100);"Bacteroidetes"(100);Flavobacteria(100);"Flavobacteriales"(100);Flavobacteriaceae(100);Cloacibacterium(100);
seed	2.03372	0.004678	4	0.035571	Bacteria(100);"Proteobacteria"(100);Betaproteobacteria(100);Rhodocyclales(100);Rhodocyclaceae(100);Sulfuritalea(100);
seed	2.06732	3.86E-05	3	0.026679	Bacteria(100);"Bacteroidetes"(100);Flavobacteria(100);"Flavobacteriales"(100);Flavobacteriaceae(100);Cloacibacterium(100);
seed	2.07038	3.86E-05	3	0.026679	Bacteria(100);"Proteobacteria"(100);Betaproteobacteria(100);Burkholderiales(100);Comamonadaceae(100);Acidovorax(100);
seed	2.0714	3.86E-05	3	0.026679	Bacteria(100);"Proteobacteria"(100);Gammaproteobacteria(100);"Enterobacteriales"(100);Enterobacteriaceae(100);Escherichia_Shigella(100);
seed	2.13194	0.004678	3	0.026679	Bacteria(100);"Proteobacteria"(100);Gammaproteobacteria(100);Pseudomonadales(100);Moraxellaceae(100);Acinetobacter(100);
seed	2.13088	0.004678	3	0.026679	Bacteria(100);"Proteobacteria"(100);Gammaproteobacteria(100);"Enterobacteriales"(100);Enterobacteriaceae(100);Escherichia_Shigella(100);
seed	2.0181	0.00118	3	0.026679	Bacteria(100);"Proteobacteria"(100);Gammaproteobacteria(100);"Enterobacteriales"(100);Enterobacteriaceae(100);Escherichia_Shigella(100);
seed	2.06974	3.86E-05	3	0.026679	Bacteria(100);"Proteobacteria"(100);Gammaproteobacteria(100);"Enterobacteriales"(100);Enterobacteriaceae(100);Escherichia_Shigella(100);
seed	2.13054	0.004678	3	0.026679	Bacteria(100);"Proteobacteria"(100);Gammaproteobacteria(100);"Enterobacteriales"(100);Enterobacteriaceae(100);Salmonella(100);
seed	2.12953	0.004678	3	0.026679	Bacteria(100);"Proteobacteria"(100);Gammaproteobacteria(100);Pseudomonadales(100);Moraxellaceae(100);Acinetobacter(100);
seed	2.06823	3.86E-05	3	0.026679	Bacteria(100);"Proteobacteria"(100);Gammaproteobacteria(100);"Enterobacteriales"(100);Enterobacteriaceae(100);Salmonella(100);
seed	2.05961	3.86E-05	3	0.026679	Bacteria(100);"Proteobacteria"(100);Betaproteobacteria(100);Burkholderiales(100);Comamonadaceae(100);Diaphorobacter(100);
seed	2.04282	3.86E-05	3	0.026679	Bacteria(100);"Proteobacteria"(100);Gammaproteobacteria(100);"Enterobacteriales"(100);Enterobacteriaceae(100);Escherichia_Shigella(100);
seed	2.10991	3.86E-05	3	0.026679	Bacteria(100);"Proteobacteria"(100);Alphaproteobacteria(100);Rhizobiales(100);Methylocystaceae(100);Methylocystis(100);
seed	2.05238	3.86E-05	3	0.026679	Bacteria(100);"Bacteroidetes"(100);Flavobacteria(100);"Flavobacteriales"(100);Flavobacteriaceae(100);Cloacibacterium(100);
seed	2.13169	0.004678	3	0.026679	Bacteria(100);"Proteobacteria"(100);Betaproteobacteria(100);Burkholderiales(100);Comamonadaceae(100);Acidovorax(100);
seed	2.08074	3.86E-05	3	0.026679	Bacteria(100);"Proteobacteria"(100);Betaproteobacteria(100);Burkholderiales(100);Comamonadaceae(100);Acidovorax(100);
seed	2.13229	0.004678	3	0.026679	Bacteria(100);"Proteobacteria"(100);Gammaproteobacteria(100);"Enterobacteriales"(100);Enterobacteriaceae(100);Escherichia_Shigella(100);
seed	2.13167	0.004678	3	0.026679	Bacteria(100);"Proteobacteria"(100);Gammaproteobacteria(100);"Enterobacteriales"(100);Enterobacteriaceae(100);Escherichia_Shigella(100);
seed	2.13149	0.004678	3	0.026679	Bacteria(100);"Proteobacteria"(100);Gammaproteobacteria(100);Pseudomonadales(100);Moraxellaceae(100);Acinetobacter(100);
seed	2.13156	0.004678	3	0.026679	Bacteria(100);"Proteobacteria"(100);Gammaproteobacteria(100);"Enterobacteriales"(100);Enterobacteriaceae(100);Escherichia_Shigella(100);
seed	2.08911	3.86E-05	3	0.026679	Bacteria(100);"Bacteroidetes"(100);Flavobacteria(100);"Flavobacteriales"(100);Flavobacteriaceae(100);Cloacibacterium(100);
seed	2.07589	3.86E-05	3	0.026679	Bacteria(100);"Bacteroidetes"(100);Flavobacteria(100);"Flavobacteriales"(100);Flavobacteriaceae(100);Cloacibacterium(100);
seed	2.13005	0.004678	3	0.026679	Bacteria(100);"Proteobacteria"(100);Betaproteobacteria(100);Burkholderiales(100);Burkholderiales_incertae_sedis(100);Aquabacterium(100);
seed	2.06765	3.86E-05	3	0.026679	Bacteria(100);"Bacteroidetes"(100);Flavobacteria(100);"Flavobacteriales"(100);Flavobacteriaceae(100);Cloacibacterium(100);
seed	2.01669	0.00118	3	0.026679	Bacteria(100);"Proteobacteria"(100);Betaproteobacteria(100);Burkholderiales(100);Comamonadaceae(100);Acidovorax(100);
seed	2.06172	3.86E-05	3	0.026679	Bacteria(100);"Proteobacteria"(100);Gammaproteobacteria(100);"Enterobacteriales"(100);Enterobacteriaceae(100);Escherichia_Shigella(100);
seed	2.07297	3.86E-05	3	0.026679	Bacteria(100);"Proteobacteria"(100);Betaproteobacteria(100);Burkholderiales(100);Comamonadaceae(100);Acidovorax(100);
seed	2.0178	0.00118	3	0.026679	Bacteria(100);"Proteobacteria"(100);Gammaproteobacteria(100);Legionellales(100);Legionellaceae(100);Legionella(100);
seed	2.06137	3.86E-05	3	0.026679	Bacteria(100);"Proteobacteria"(100);Gammaproteobacteria(100);"Enterobacteriales"(100);Enterobacteriaceae(100);Escherichia_Shigella(100);

reactor	LDA	pValue	Size	%	Taxonomy
reactor	4.91096	0.024589	35484	35.72155	Bacteria(100);"Proteobacteria"(100);Alphaproteobacteria(100);Rhizobiales(100);Bradyrhizobiaceae(100);Bradyrhizobium(100);
reactor	4.62189	0.024515	14186	14.28097	Bacteria(100);"Proteobacteria"(98);Alphaproteobacteria(79);Rhodospirillales(79);Rhodospirillaceae(79);Telmatospirillum(79);
reactor	4.59897	0.024515	11668	11.74611	Bacteria(100);"Proteobacteria"(100);"Zetaproteobacteria"(94);"Mariprofundales"(94);"Mariprofundaceae"(94);Mariprofundus(94);
reactor	4.4791	0.024515	8822	8.881059	Bacteria(100);"Proteobacteria"(100);Alphaproteobacteria(100);Rhizobiales(100);Hyphomicrobiaceae(100);Hyphomicrobium(100);
reactor	4.12477	0.024515	4040	4.067046	Bacteria(100);"Actinobacteria"(100);Actinobacteria(100);Actinomycetales(100);Microbacteriaceae(100);Microbacterium(100);
reactor	4.14169	0.034714	3776	3.801279	Bacteria(100);"Proteobacteria"(100);Alphaproteobacteria(100);Rhizobiales(100);Bradyrhizobiaceae(100);Bradyrhizobium(100);
reactor	4.11292	0.024515	3683	3.707656	Bacteria(100);"Proteobacteria"(100);Alphaproteobacteria(100);Rhizobiales(100);Hyphomicrobiaceae(100);Hyphomicrobium(100);
reactor	4.03438	0.024515	3295	3.317058	Bacteria(100);"Proteobacteria"(100);Alphaproteobacteria(100);Rhizobiales(100);Hyphomicrobiaceae(100);Hyphomicrobium(100);
reactor	4.01476	0.034714	3132	3.152967	Bacteria(100);"Proteobacteria"(98);Alphaproteobacteria(86);Rhodospirillales(86);Rhodospirillaceae(86);Telmatospirillum(86);
reactor	3.95801	0.034714	2862	2.88116	Bacteria(100);"Proteobacteria"(100);Alphaproteobacteria(100);Rhodospirillales(93);Rhodospirillaceae(93);Dongia(93);
reactor	3.74479	0.034714	1636	1.646952	Bacteria(100);"Proteobacteria"(100);Alphaproteobacteria(100);Sphingomonadales(100);Sphingomonadaceae(100);Novosphingobium(100);
reactor	3.72264	0.024515	1559	1.569437	Bacteria(100);"Proteobacteria"(100);Alphaproteobacteria(100);Rhizobiales(100);Hyphomicrobiaceae(92);Devosia(92);
reactor	3.57954	0.048035	1205	1.213067	Bacteria(100);"Bacteroidetes"(100);"Sphingobacteria"(100);"Sphingobacteriales"(100);Chitinophagaceae(100);Sediminibacterium(96);
reactor	3.56756	0.034714	1136	1.143605	Bacteria(100);"Acidobacteria"(100);Holophagae(100);Holophagales(100);Holophagaceae(100);Geothrix(100);
reactor	3.2942	0.048035	608	0.61207	Bacteria(100);"Proteobacteria"(100);Alphaproteobacteria(100);Caulobacterales(100);Caulobacteraceae(100);Brevundimonas(100);
reactor	3.1693	0.048035	428	0.430865	Bacteria(100);"Proteobacteria"(100);Alphaproteobacteria(100);Rhizobiales(100);Phyllobacteriaceae(100);Mesorhizobium(100);
reactor	3.08138	0.048035	364	0.366437	Bacteria(100);"Proteobacteria"(100);Alphaproteobacteria(100);Rhizobiales(100);Phyllobacteriaceae(100);Pseudaminobacter(100);
reactor	2.94832	0.034714	279	0.280868	Bacteria(100);"Proteobacteria"(100);Alphaproteobacteria(100);Rhodobacterales(100);Rhodobacteraceae(100);Gemmobacter(98);
reactor	3.03089	0.048035	275	0.276841	Bacteria(100);"Proteobacteria"(100);Alphaproteobacteria(100);Rhizobiales(99);Beijerinckiaceae(99);Chelatococcus(98);
reactor	2.76249	0.048035	178	0.179192	Bacteria(100);"Proteobacteria"(100);Alphaproteobacteria(100);Rhodospirillales(100);Acetobacteraceae(100);Roseomonas(77);
reactor	2.71951	0.034714	164	0.165098	Bacteria(100);Firmicutes(88);Negativicutes(88);Selenomonadales(88);Veillonellaceae(88);Anaerosinus(88);
reactor	2.57482	0.03509	133	0.13389	Bacteria(100);"Proteobacteria"(100);Alphaproteobacteria(100);Rhizobiales(100);Bradyrhizobiaceae(100);Bradyrhizobium(100);
reactor	2.4391	0.048035	80	0.080536	Bacteria(100);"Proteobacteria"(100);Gammaproteobacteria(100);Pseudomonadales(100);Pseudomonadaceae(100);Pseudomonas(100);
reactor	2.34315	0.03509	79	0.079529	Bacteria(100);"Proteobacteria"(100);Alphaproteobacteria(100);Rhizobiales(100);Bradyrhizobiaceae(100);Bradyrhizobium(100);
reactor	2.26717	0.024515	48	0.048321	Bacteria(100);"Proteobacteria"(100);Alphaproteobacteria(100);Rhizobiales(100);Bradyrhizobiaceae(100);Bradyrhizobium(100);
reactor	2.194	0.048035	43	0.043288	Bacteria(100);"Proteobacteria"(100);Alphaproteobacteria(100);Rhizobiales(100);Bradyrhizobiaceae(100);Bradyrhizobium(100);
reactor	2.14073	0.024515	37	0.037248	Bacteria(100);"Proteobacteria"(100);Alphaproteobacteria(100);Rhizobiales(100);Bradyrhizobiaceae(100);Bradyrhizobium(100);
reactor	2.07748	0.048035	36	0.036241	Bacteria(100);"Proteobacteria"(100);Alphaproteobacteria(100);Rhizobiales(100);Bradyrhizobiaceae(100);Bradyrhizobium(100);
reactor	2.0849	0.048035	36	0.036241	Bacteria(100);"Proteobacteria"(100);Alphaproteobacteria(100);Rhizobiales(100);Bradyrhizobiaceae(100);Bradyrhizobium(100);
reactor	2.09393	0.048035	32	0.032214	Bacteria(100);"Proteobacteria"(100);Alphaproteobacteria(100);Rhizobiales(100);Bradyrhizobiaceae(100);Bradyrhizobium(100);
reactor	2.04365	0.048035	31	0.031208	Bacteria(100);"Proteobacteria"(100);Alphaproteobacteria(100);Rhizobiales(100);Bradyrhizobiaceae(100);Bradyrhizobium(100);
